# Quantitative imaging of membrane contact sites for sterol transfer between endo-lysosomes and mitochondria in living cells

**DOI:** 10.1038/s41598-021-87876-7

**Published:** 2021-04-26

**Authors:** Alice Dupont Juhl, Christian W. Heegaard, Stephan Werner, Gerd Schneider, Kathiresan Krishnan, Douglas F. Covey, Daniel Wüstner

**Affiliations:** 1 Department of Biochemistry and Molecular Biology, VILLUM Center for Bioanalytical Sciences, University of Southern Denmark, Campusvej 55, 5230 Odense M, Denmark; 2 Department of Molecular Biology and Genetics, University of Aarhus, 8000 Aarhus C, Denmark; 3 Department of X-Ray Microscopy, Helmholtz-Zentrum Berlin, Albert-Einstein-Str. 15, 12489 Berlin, Germany; 4 Department of Developmental Biology, Washington University, St. Louis, MO 63110 USA

**Keywords:** Biophysical chemistry, Lipids, Cellular imaging, Data acquisition, Data processing, Image processing, Machine learning, Statistical methods, Lysosomes, Multivesicular bodies, Lysosomes, Mitochondria, Multivesicular bodies

## Abstract

Mitochondria receive cholesterol from late endosomes and lysosomes (LE/LYSs) or from the plasma membrane for production of oxysterols and steroid hormones. This process depends on the endo-lysosomal sterol transfer protein Niemann Pick C2 (NPC2). Using the intrinsically fluorescent cholesterol analog, cholestatrienol, we directly observe sterol transport to mitochondria in fibroblasts upon treating NPC2 deficient human fibroblasts with NPC2 protein. Soft X-ray tomography reveals the ultrastructure of mitochondria and discloses close contact to endosome-like organelles. Using fluorescence microscopy, we localize endo-lysosomes containing NPC2 relative to mitochondria based on the Euclidian distance transform and use statistical inference to show that about 30% of such LE/LYSs are in contact to mitochondria in human fibroblasts. Using Markov Chain Monte Carlo image simulations, we show that interaction between both organelle types, a defining feature of membrane contact sites (MCSs) can give rise to the observed spatial organelle distribution. We devise a protocol to determine the surface fraction of endo-lysosomes in contact with mitochondria and show that this fraction does not depend on functional NPC1 or NPC2 proteins. Finally, we localize MCSs between LE/LYSs containing NPC2 and mitochondria in time-lapse image sequences and show that they either form transiently or remain stable for tens of seconds. Lasting MCSs between endo-lysosomes containing NPC2 and mitochondria move by slow anomalous sub-diffusion, providing location and time for sterol transport between both organelles. Our quantitative imaging strategy will be of high value for characterizing the dynamics and function of MCSs between various organelles in living cells.

## Introduction

Cholesterol is an important and abundant lipid which regulates the biophysical properties of cellular membranes including their fluidity, curvature, permeability and protein-lipid interactions^1,2^. Several trafficking pathways work in concert to maintain the cholesterol content of cellular membranes within their characteristic range, which varies greatly between the plasma membrane (PM), endosomes, lysosomes, Golgi apparatus, ER and mitochondria^2,3^. The majority of cellular cholesterol is found in the PM and some endosome populations, which together contain approximately 70–80% of cellular cholesterol compared to about 5% in the ER and mitochondria^2,3^. Cholesterol trafficking between sterol-rich organelles, such as the PM and endosomes and metabolically active but sterol-poor organelles, like ER and mitochondria, is very important, as the two latter harbor the molecular machinery to sense cellular cholesterol abundance and to convert cholesterol into essential sterol metabolites, respectively^4^. Continuous delivery of a portion of cellular cholesterol to mitochondria ensures synthesis of cholesterol-derived sterols, like oxysterols and steroid hormones via mitochondria-localized oxidoreductases. The availability of cholesterol constitutes the rate-limiting step of steroidogenesis and is regulated by STARD1 which transfers cholesterol from the outer to the inner mitochondrial membrane^5^. Both mitochondrial membranes require cholesterol for membrane maintenance, and as consequence of the low sterol content, even small changes in the cholesterol concentration can have a relatively large impact on these membranes^2,6^. Increased cholesterol concentration of mitochondria can cause reduced fluidity of their membranes, reduced ATP generation and decreased import of the key survival antioxidant; mitochondrial glutathione^7,8^. When cellular cholesterol trafficking is interrupted, it can lead to fatal disorders, such as the neurodegenerative Niemann Pick type C (NPC) disease, in which cholesterol accumulates in late endosomes and lysosomes (LE/LYSs) and fails to reach the homeostatic sensing machinery in the ER^4^. NPC disease is caused by dysfunction of either the NPC1 or NPC2 protein. NPC1 is a large membrane protein in the perimeter of LE/LYSs, while NPC2 is a small soluble sterol-binding protein in the lumen of LE/LYSs. NPC2 but not NPC1 has been implicated in the delivery of cholesterol from LE/LYSs to mitochondria^9^, but the mechanistic details of this transport process are not known.

Since mitochondria form contacts to endosomes and endoplasmic reticulum, an attractive hypothesis is that cholesterol transport to mitochondria takes place via such membrane contact sites (MCSs)^2,10^. However, whether MCSs form between endo-lysosomes containing NPC2 and mitochondria is not known. Also, sterol transport to mitochondria has not been directly observed in a live-cell imaging setup. Here, we explore the possibility, that MCSs form between LE/LYSs and mitochondria to ensure cholesterol transport between both organelles. We use ultraviolet (UV) sensitive microscopy of an intrinsically fluorescent analog of cholesterol, cholestatrienol (CTL), which differs from cholesterol only by having two additional double bonds in the steroid ring system, to show that sterol transfer to mitochondria requires the NPC2 protein. Next, we present new image analysis protocols to quantify the extent and dynamics of formation of MCSs between endo-lysosomes containing NPC2 and mitochondria. We show that distances between both types of organelles can be automatically quantified by combining single particle tracking (SPT) and 3D organelle segmentation with calculation of the Euclidian distance transform of binarized mitochondria images. Employing a Gaussian mixture model to the measured organelle distance distribution allows identification of a subpopulation of endo-lysosomes that are in contact with mitochondria. Using a Markov Chain Monte Carlo (MCMC) simulation procedure, we assess interactions between LE/LYSs and mitochondria directly from microscope images. By SPT of MCSs between NPC2 containing LE/LYSs and mitochondria over time, we demonstrate that MCSs form dynamic clusters within small areas of confinement and slow anomalous sub-diffusion. We suggest that such MCSs provide a pathway for cholesterol transport between both organelles.

See Tables 1, 2, 3, 4, 5.

**Table 1:** Summary of *A. senhousia* population data from sites within the Solent region of the UK, recorded from 2007–2019. Site numbers correlate with Fig. 1. Gonad stages are based on those of Sgro et al.^19^: “1–2” = spent or developing; “3–4” = ripe or spawning; “–” = data not collected

Habitat	Location	Site	Year	Count	Density (m^−2^): Range, mean, +/− SD	Shell length (mm): Range, mean, +/− SD	Gonad stage	Surveying organisation
Subtidal	Southampton Water	1–45	2007	0	–	–	–	Environment Agency
		46–70	2011	18	0–70, 7.2 +/− 18.6	–	–	
		71–95	2013	10	0–70, 4 +/− 14.1	–	–	
		96–120	2016	51	0–290, 20.4 +/− 58.8	–	–	
Intertidal	Hythe, River Test	121	2016	1	–	17	–	Pisces Conservation Ltd
			2019	1	–	18	–	
Intertidal	Brownwich	122	2017	5	–	14.1–20.8, 17.9 +/− 2.5	–	University of Portsmouth
			2019	169	0.06	9–32, 20.1 +/− 3.9	March–May: 1–2; July: 3–4; Sept/Oct: 1–4 (in 2019)	
Intertidal	Weston Shore, River Itchen	123	2018	2	–	–	–	University of Southampton
Marina; suspended hard surfaces	Saxon Wharf, River Itchen	124	2018	3	0.67	7–13, 8.7 +/− 3.1	–	University of Portsmouth
			2018	14	–	13–23, 17.6 +/− 3.0	–	
Marina; suspended hard surfaces	Port Hamble, River Hamble	125	2019	–	–	–	–	University of Portsmouth
Intertidal	Lepe	126	2019	1	–	–	–	Hampshire and Isle of Wight Wildlife Trust volunteer
Intertidal; *Zostera marina*, *Z. noltei* beds	Portsmouth Harbour	127	2019	1	4	18	–	University of Portsmouth
Intertidal; highly sheltered	Chichester Harbour	128	2019	1	–	4	–	University of Portsmouth
Marina; suspended hard surfaces	Shamrock Quay, River Itchen	129	2019	2	–	19–28	–	University of Portsmouth
Subtidal	Newtown, Isle of Wight	130	2019	1	–	21	–	University of Portsmouth

**Table 2:** A summary of the impacts of *A. senhousia* in relation to Provisioning ecosystem services. (+) denotes a potentially positive impact, (–) denotes a potentially negative impact. Priority questions are those that should be addressed by researchers to generate a full risk assessment and management plan

Provisioning ecosystem services	+/−	*A. senhousia* impacts and observations	+/−	Supporting information	+/−	Priority questions
Food (wild, farmed)	–	Biofouling organism. Attached to *O.edulis* and concrete plates (this study); Hong Kong oyster (*Crassostrea hongkongensis*)^37^; synthetic capron line (126,000 spat/m^2^)^3^	–	Spawning may overlap with *Mytilus* spp. in Europe: *A. senhousia* spawning prolonged in introduced range^1,19,24,56,63,97^; gonad ripening by July in UK (this study); documented hybridisation amongst *Mytilus* spp.^91,97–99^	–	Disrupts the cultivation of commercial species through biofouling (i.e. more intense cleaning required)?
					–	Disrupts the cultivation of commercial species through resource competition?
	–	Reduces clam (*Chione* spp., *Mactra* spp., *Meretrix lusoria*, *Ruditapes philippinarum*) growth and survivorship via space and food competition and by increasing predation^70–73^			–	Introduces diseases which impact commercial species?
			–	Introduced bivalve molluscs can facilitate the spread of shellfish diseases^90^	–	Hybridises with commercial and native species, influencing genetic diversity?
	**+**	Human consumption in China^22,96^			+/−	Consumed by people in introduced range?
Animal feed (wild, farmed, bait)	**+**	Fish bait and feed stock for shrimp and crab aquaculture in Japan^21^	**+**	Mollusc shells used as poultry grit^95,100^	**+**	Use as poultry grit?
Pet trade products		–	**+**	Mollusc shells used for pet bird nutrition and aquarium pH buffer^95,100^	**+**	Use as pet bird nutrition and aquarium pH buffer?
Fertilizer		–	**+**	Mollusc shells used as soil conditioner^95,100^	**+**	Use as soil conditioner?
Aggregates extraction		–	**+**	Mollusc shells are used for: construction materials; biofilter medium; calcium acetate road de–icer^95,100^	**+**	Use as: construction materials; biofilter medium; calcium acetate road de-icer?

**Table 3:** A summary of the impacts of *A. senhousia* in relation to Regulating ecosystem services. (+) denotes a potentially positive impact, (–) denotes a potentially negative impact. Priority questions are those that should be addressed by researchers to generate a full risk assessment and management plan

Regulating ecosystem services	+/−	*A. senhousia* impacts and observations	+/−	Supporting information	+/−	Priority questions
Waste (excess nutrients, toxic pollutants) remediation	**+**	Removes excess nitrogen and phosphorus from water^101^ (excess nutrients are detrimental to *Zostera* spp)^85^	**+**	Mussels such as *M. edulis* sequester and store toxic pollutants (mutagenic/ carcinogenic hydrocarbons, heavy metals, micro plastics, nanoparticles, pharmaceuticals)^102^	**+**	Nutrient remediation (nitrogen and phosphorus): reduction in size/frequency of eutrophication events and harmful algal blooms (HABs)?
					**+**	Reduction of toxic pollutants in pelagic zone?
Natural hazard protection	**+**	*Arcuatula senhousia* mats can stabilise soft sediments^40^ likely reducing resuspension events^103^	**+**	Mussel mats offer protection of ecologically sensitive habitats such as seagrass beds and salt marshes by reducing shoreline and bed erosion^100^	**+**	Mats work as coastal sea defences?
					**+**	Mats reduce resuspension events?
Climate regulation	–	An additional source of CO_2_ in seawater, increasing CO_2_ evasion from seawater into the atmosphere^104^	+/−	Bivalves can influence the carbon budget via calcification: sequestration of carbon in the form of calcium carbonate and the release of carbon in the form of CO_2_^100,105^	+/−	Carbon source or sink?

**Table 4:** A summary of the impacts of *A. senhousia* in relation to Supporting ecosystem services. (+) denotes a potentially positive impact, (–) denotes a potentially negative impact. Priority questions are those that should be addressed by researchers to generate a full risk assessment and management plan

Supporting ecosystem services		*A. senhousia* impacts and observations		Supporting information		Priority questions
Provision of habitat	–	Attached to native European flat oyster (*O. edulis*) shells and roof tiles used for its cultivation where there are efforts to restore *O. edulis* populations (this study)	–	Introduction of commercial bivalve molluscs such as *Magallana gigas* can introduce non–native epifauna that hitch–hike on shells^51^	–	Interferes with native shellfish (e.g. *O. edulis*) restoration?
					+/−	Inhibits or facilitates seagrass (e.g. *Zostera* spp.) beds?
	+/−	Inhibitive and potentially facilitative effects on seagrass (*Zostera marina*)^34,36,68^*.*			+/−	Outcompetes other invasive species?
			**+**	Mussels such as *M. edulis* facilitate removal of fine sediment from the pelagic zone^41,109^. Likely true for *A. senohousia* since levels of fine sediment are higher within mats^40^	–	Introduces non-native shell epifauna?
	+/−	Directly settle on *Zostera* blades as juveniles^79^ – probably later become dislodged^106,107^. Found within beds of *Z. marina* and *Z. noltei* (this study). Causes changes in macrobenthos species community^23,40,63,108^			–	Creates habitat for other invasive species?
					**+**	Reduces smothering of benthic fauna by fine sediment?
Provision of food	+/−	Food source for predators: birds (diving ducks and oyster catchers)^8,63,67^, boring gastropods^52,68,69^, fish^110^ and probably crustaceans and echinoderms due to its thin shell		–	+/−	Causes changes in dispersal patterns and/or numbers of predators
Genetic diversity		–	–	Potential for hybridisation with native species (see row 1.2.)	–	Hybridises with commercial and native species, influencing genetic diversity?

**Table 5:** A summary of the impacts of *A. senhousia* in relation to Cultural ecosystem services. (+) denotes a potentially positive impact, (–) denotes a potentially negative impact. Priority questions are those that should be addressed by researchers to generate a full risk assessment and management plan

Cultural ecosystem services	+/−	*A. senhousia* impacts and observations	+/−	Supporting information	+/−	Priority questions
Recreation	–	Provides habitat and food for a toxic sea slug (*Pleurobranchaea maculata*) in New Zealand which is harmful to dogs^111^	+/−	Introduced molluscs such as *M. gigas* can provide feeding grounds for some shorebird spp. but destroy it for others^112^	+/−	Impacts bird watching?
					**+**	Reduces *Escherichia coli* in bathing waters?
	–	Biofouling organism. Found attached to boat hulls^52^	**+**	Mussels such as *M. edulis* sequester and store toxic pollutants^102^	–	Increases time and money spent on cleaning boat hulls?
Visual amenity	+/−	Forms large mats on soft sediment and can attach to hard surfaces^1,34,101,113^ (this study)		–	+/−	Changes aesthetics of marine infrastructure and beaches?
Human health		–	–	Human enteric viruses are carried by cultured and wild mussels^114^	–	Carries bacteria or viruses harmful to humans?

## Results

### NPC2 in LE/LYSs is necessary for transport of sterol from the PM to mitochondria

**Figure 1:**
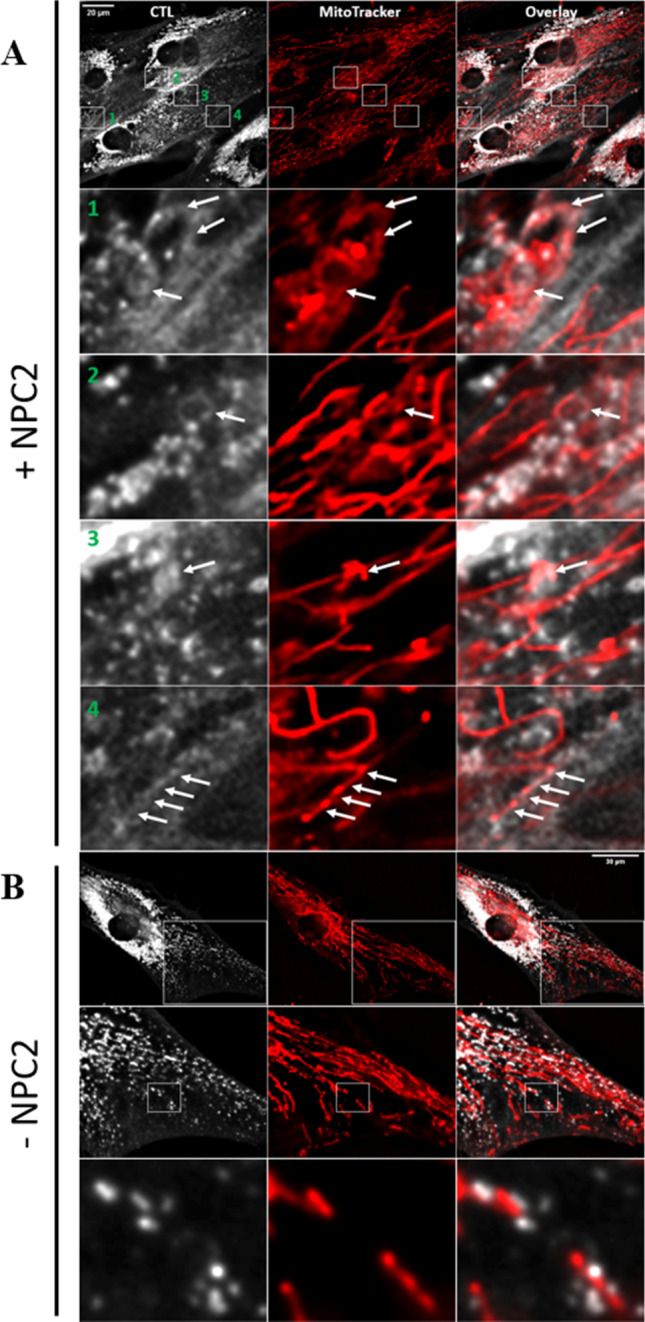
Direct observation of sterol transport to mitochondria. Human fibroblasts of a NPC2-disease patient (NPC2−/− fibroblasts) were loaded with 20 μM CTL, from a CTL/BSA complex for 48 h in LPDS medium and subsequently chased for 24 h in LPDS medium with (**A**) or without (**B**) 200 nM NPC2 protein, and co-stained with MitoTracker green before imaging on an UV sensitive wide field microscope. Arrows point to CTL in mitochondria. Insets in panel **A** (numbered 1–4) are shown as zooms below the first row. The inset in the upper row of panel **B** is shown as zoom underneath in which another inset is indicated as white box and zoomed as lowest row. Bar, 20 µm.

Current evidence for NPC2’s role in cholesterol transport from LE/LYSs to mitochondria relies on biochemical assays, in which formation of steroid hormone precursors, such as pregnenolone from cholesterol is monitored, a reaction, which takes place in mitochondria^9^. We aim for a direct visualization of sterol transport to mitochondria in living cells, but this is challenging, as the cholesterol content of mitochondria is very low. Also, we want to avoid using tagged cholesterol analogues, which can artifactually accumulate in certain organelles, including mitochondria and lipid droplets^11,12^. Instead, we employed the close cholesterol analogue CTL, which contains only two additional double bonds compared to cholesterol giving CTL a slight fluorescence in the ultraviolet (UV; see Fig. S1A for structures). In human fibroblasts lacking functional NPC2 (NPC2−/− cells), CTL accumulates in LE/LYSs after uptake from the PM^13^. When incubating those cells with NPC2 protein purified from bovine milk, we can rescue the lysosomal sterol storage phenotype^13,14^. Co-staining those cells with MitoTracker, we find close apposition of endo-lysosomes containing CTL and mitochondria and frequently faint staining of mitochondria (Fig. 1A). We would not expect more than faint labeling, as the cholesterol content of mitochondria is much lower than that of LE/LYSs, and CTL is a comparably dim but reliable fluorescent cholesterol probe. Importantly, in NPC2-deficient cells not being treated with NPC2 protein, close apposition between CTL containing LE/LYSs and mitochondria was still found, but no CTL in mitochondria could be detected (Fig. 1B). In contrast, CTL was observed in fibroblasts from healthy subjects and from patients lacking functional NPC1 protein (Fig. S1B and C). Together, these results support earlier findings that NPC2 but not NPC1 is needed for cholesterol transport from endo-lysosomes to mitochondria^9,15^.

### Mitochondrial ultrastructure is not altered in NPC disease fibroblasts

**Figure 2:**
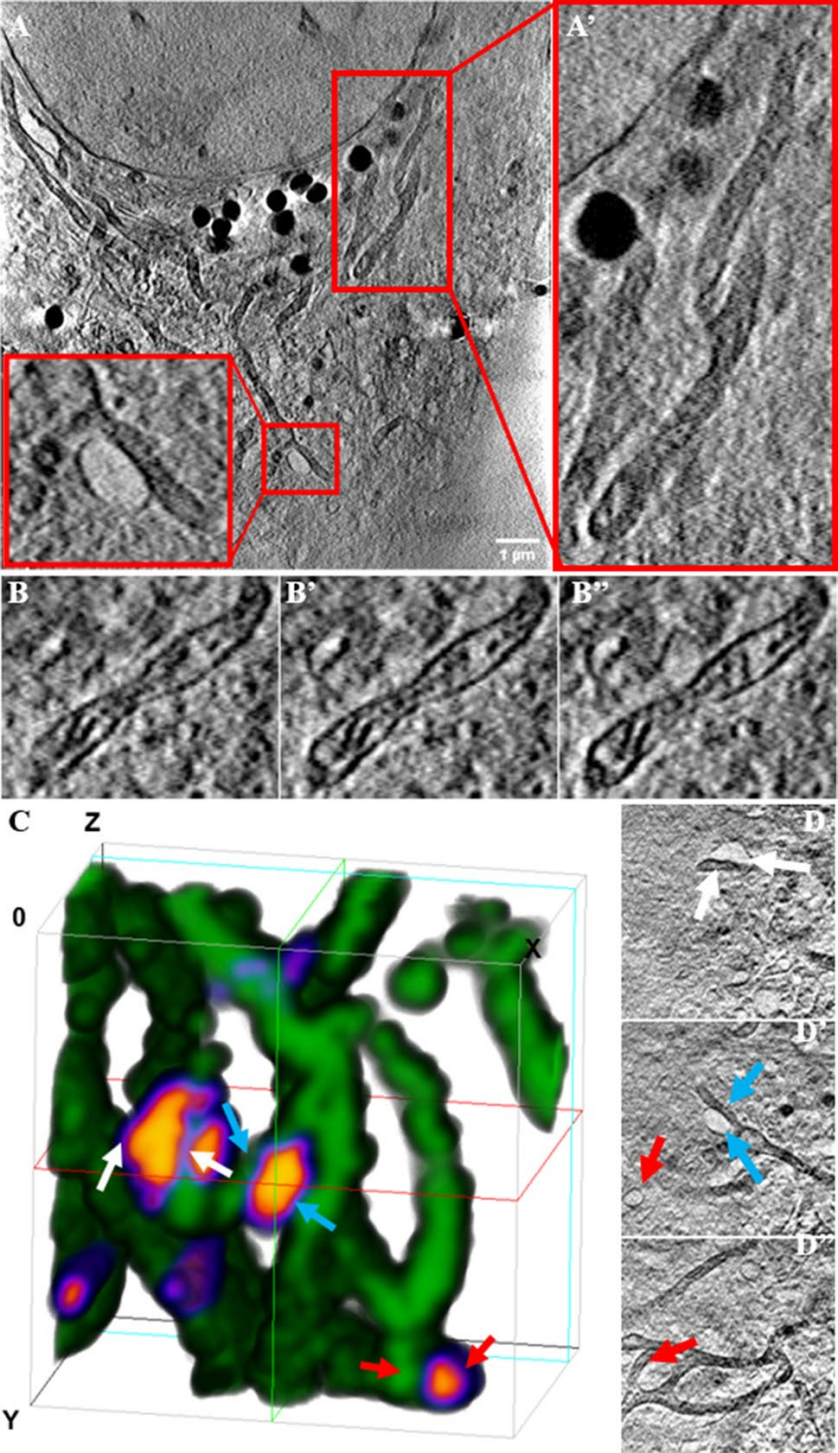
Soft X-ray tomography (SXT) reveals the ultrastructure of mitochondria and shows contacts to endosomes. NPC2−/− fibroblasts were treated with 200 nM NPC2 for 48 h, labeled with TopFluor-cholesterol, chased for 2 h and fixed with PFA. The cells were kept in PBS until plunge freezing in liquid ethane followed by imaging of tomograms at the X-ray microscope, spatial registration and 3D reconstruction of tomograms (see Materials and Methods for details). **A**–**A**’, right red box and zoom highlight a branched mitochondrion, while lower left box and zoom point to a contact area between a mitochondrion and an endosome-like vesicle. **B**, sections though another mitochondrion reveals its ultrastructure with clearly discernable cristae. **C**–**D**’’, 3D rendering of the reconstructed tomogram shown in D. Different colored arrows in **D**, **D**’, **D**’’ point to endosome-like in close contact with mitochondria, the corresponding color arrows point to same structures in the rendering in C. Bar, 1 μm.

Mitochondrial morphology is tightly regulated by controlled fusion and fission processes, and this balance is often altered under starvation and certain disease conditions^16^. To assess, whether the NPC mutations change the length and morphology of mitochondria, we quantified these parameters from fluorescence images using the Mitochondria Network Analysis (MiNA) toolset^17^. We found that the length of mitochondria and their extent of branching was higher in fibroblasts from NPC patients compared to cells from healthy control subjects (Fig. S2). This points to a more fused state of mitochondria in NPC disease cells, an indication of oxidative and other metabolic stress conditions. Mitochondria of cells from NPC2 patients were slightly more elongated and branched compared to those of NPC1 patients but treating the former fibroblasts with NPC2 protein did not reverse this phenotype (Fig. S2). To assess the ultrastructure of mitochondria, we employed soft X-ray tomography (SXT). SXT is a label-free imaging technique, achieving isotropic resolution of 30–50 nm in three dimensions throughout an entire cellular volume by absorption of X-rays^18^. Using SXT, we observe extensively branched mitochondria in NPC2−/− fibroblasts, in which intramitochondrial cisternae could be clearly discerned (Fig. 2A and B). No difference in mitochondrial ultrastructure was found between NPC2-treated and untreated NPC2−/− fibroblasts (Fig. 2B and S3). We observed repeatedly that some mitochondria form close contacts to endosome-like organelles, which become partly enwrapped by the mitochondrial network with inter-organelle distances of less than 50 nm (Fig. 2C and D and Fig. S3A and B). This could resemble MCSs between both organelle types, which might allow for exchange of cholesterol, metabolites, or ions.

### A population of endo-lysosomes containing NPC2 are in contact with mitochondria

**Figure 3:**
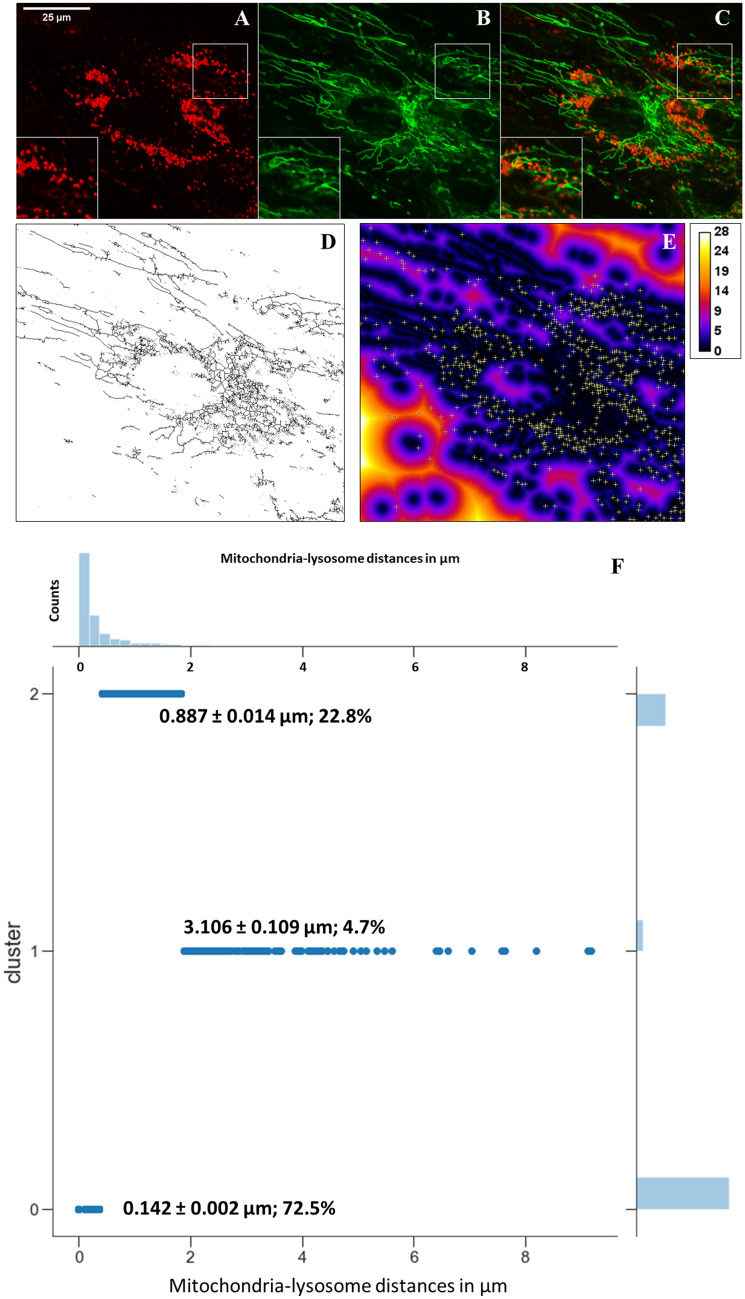
Automated quantification of distances between LE/LYSs and mitochondria. NPC2 −/− fibroblasts were incubated with 100 nM Alexa546-NPC2 for 72 h in LPDS medium and labeled for 30 min with MitoTracker Green before imaging at a spinning disk confocal microscope. A-C, single confocal sections showing Alexa546-NPC2 (**A**), MitoTracker (**B**) and Overlay (**C**). The skeletonized version of the MitoTracker image (**D**) was used to calculate the Euclidian distance transform (EDT), onto which the position of endo-lysosomes containing Alexa546-NPC2 (from A) was mapped (yellow crosses in **E**; distance is color-coded in a FIRE LUT and given in µm). The distance distribution from six cells comprising 3560 LE/LYSs containing Alexa546-NPC2 to neighboring mitochondria was analyzed with a Gaussian mixture model to identify subpopulations, resulting in three clusters (**F**). The x-axis shows the mitochondria-lysosome distance in µm. The y-axis of the main plot shows the three identified cluster populations, while that of the upper histogram shows the number of counts. The largest cluster (# 0) comprises 72.5% of all identified endo-lysosome/distances with a mean value of 142 nm. Bar, 25 µm.

Since MCSs could provide a pathway for sterol transfer from LE/LYSs to mitochondria, we determined the spatial relationship between both organelles on a whole-cell level. For that, NPC2−/− cells were incubated with 100 nM Alexa546-NPC2 for 72 h, and mitochondria were stained with a green MitoTracker dye before imaging on a spinning disk confocal microscope. We observed extensive clustering of LE/LYSs containing fluorescent NPC2 around mitochondria (Fig. 3A–C). To quantify this observation, we developed an image analysis protocol, in which mitochondria positions were first determined by thresholding and skeletonizing followed by inversion to set selected pixels to zero (black; Fig. 3D). To that image, we apply the Euclidian distance transform (EDT; Fig. 3E), which calculates the distance for each pixel of the image to the nearest black pixel of the binary image (Fig. 3D). In a second step, we determine the positions of all endo-lysosomes containing Alexa546-NPC2 in the corresponding image, for which a particle finding algorithm as used in SPT is applied. Finally, we map the found positions onto the EDT image, thereby determining all distances of labeled LE/LYSs to their nearest mitochondria (Fig. 3E, yellow crosses). The resulting distribution of inter-organelle distances comprises the pooled distance histogram from six cells containing together 3560 endo-lysosomes (see histogram in upper part of Fig. 3F). This distribution peaks at around 0.15 µm with shoulder peaks at around 1.0 and 2.2 µm, respectively (Fig. 3F and S4A). To infer the underlying subpopulations, we first fitted the sum of three Gaussian functions to the experimental data of distances between endo-lysosomes and mitochondria (Fig. S4A). This gave a reasonable fit but suffers from the fact, that we do not know a priori, which data point belongs to which Gaussian component. To overcome this problem, we applied a Gaussian mixture model, which iteratively calculates the probability for each data point to be generated by one of the three Gaussian components of the model^19^. This analysis revealed three populations of LE/LYSs containing NPC2; the two larger ones comprise together more than 95% of all endo-lysosome/mitochondria distances, in which the average distances are 1) 0.142 µm (cluster 0) and 2) 0.887 µm (cluster 2, Fig. 3F). The third population is very small and broadly distributed with an average distance of 3.106 µm to the next mitochondrion (cluster 1, Fig. 3F). The closest pool (cluster 0) comprises 72% of all endo-lysosomes containing fluorescent NPC2. Given that the average diameter of NPC2-containing LE/LYSs in this 2D projection is 0.297 µm (Fig. S4B), some of those LE/LYSs being closer than their radius of 0.148 µm are likely in direct contact with mitochondria.

**Figure 4:**
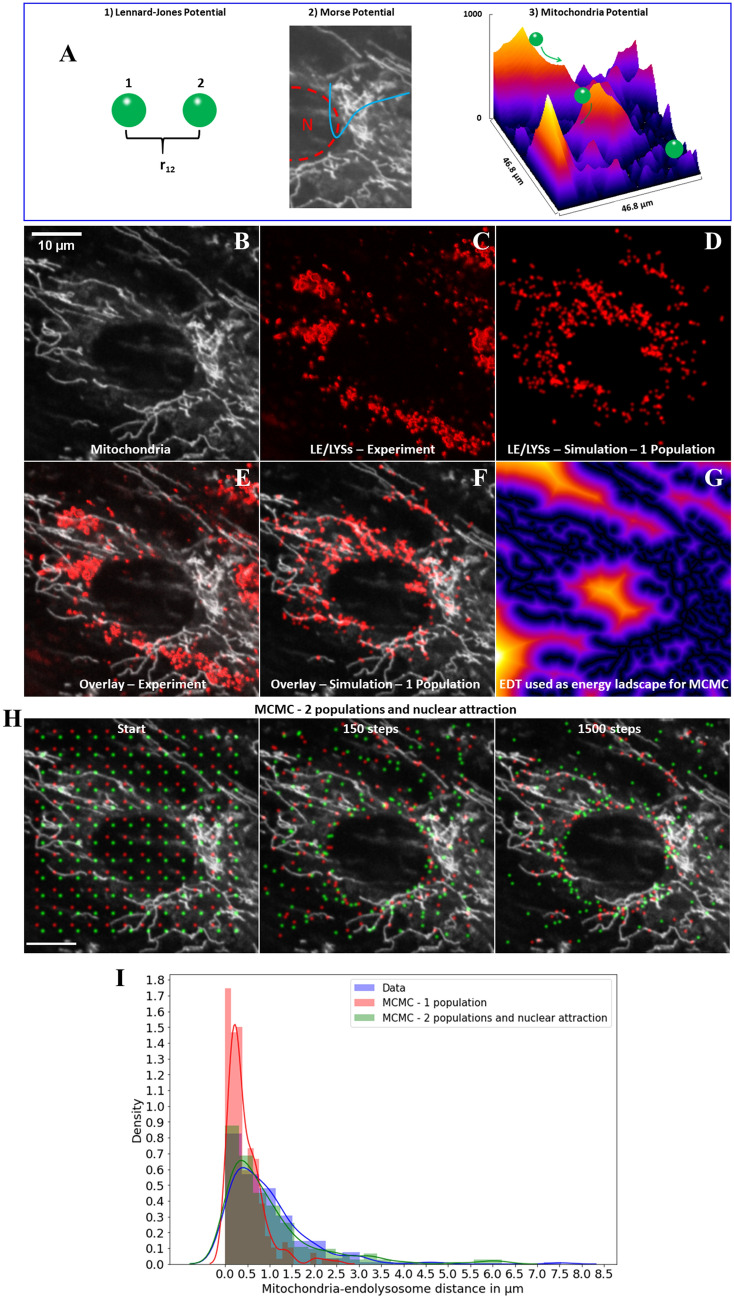
Markov Chain Monte Carlo simulation of distances between LE/LYSs and mitochondria. To simulate particles mimicking endo-lysosomes containing Alexa546-NPC2, a potential energy function was defined consisting of three contributions (**A**); (1) a Lennard Jones Potential between particles, (2) a Morse potential between particles and nucleus (middle panel, dashed line is nucleus, N; blue line indicates shape of the Morse potential) and (3) a Mitochondria potential in which attraction of LE/LYSs by mitochondria was inferred from a scaled version of the EDT map (right panel, compare G). Particles ‘roll’ into the valleys of this Mitochondria potential energy landscape to minimize their potential energy, which ensures their attraction by mitochondria. The used MitoTracker image (**B**) and corresponding experimental fluorescence image of Alexa546-NPC2 (**C**) are compared to the simulated endo-lysosome distribution for one population of particles (i.e. mimicking strong interaction) in the same cell geometry (**D**). Overlay of experimental (**E**) and simulated endo-lysosome distribution (**F**) on mitochondria image and the corresponding EDT (**G**). Clearly, experimental and simulated LE/LYSs cluster around mitochondria. **H**, snapshots of a simulation of two endo-lysosome populations + nuclear attraction. **I**, histogram of measured and simulated distances of endo-lysosomes to mitochondria from the selected cell together with a kernel density estimate of the distribution. Experimental data is in blue while simulations are shown in red and green, respectively.

The observed proximity of a portion of endo-lysosomes to mitochondria suggests some form of interaction between both organelle types. To test this notion, we set out image-based Markov Chain Monte Carlo (MCMC) simulations which can model physical interactions within the cell geometry^20^. Endosomes and lysosomes have been shown to be organized in characteristic spatial patterns, which play an important role in their function^21,22^. To model such a distribution, the spatial position of particles simulating endo-lysosomes is dictated by three types of interaction in our model; (1) a ‘Lennard Jones Potential’ between the particles, (2) an attractive potential to the nucleus modelled by a ‘Morse Potential’ to account for interaction of LE/LYSs with perinuclear microtubules^20^, and (3) an attractive potential between the endo-lysosome particles and mitochondria defining the ‘Mitochondria Potential (M-potential)’ (Fig. 4A). The Lennard Jones 6–12 potential acts between the synthetic endo-lysosomes and ensures their mutual attraction for intermediate distances (1/r^6^-dependent term) and repulsion upon contact (1/r^12^-dependent term). This type of potential energy is often employed in molecular and soft matter simulations^23^. The second term, the Morse potential is mostly used to model chemical bonds but is here employed to describe the attraction of endo-lysosomes towards the perinuclear region, as has been observed in many experiments (Fig. 4A and see Materials and Methods for details)^20,21,24,25^. The effect of adjacent mitochondria, to which simulated endo-lysosomes might bind, is defined in the M-potential which is directly inferred from the EDT map of the mitochondria image for a given cell with some scaling factor (Fig. 4A). The EDT map is taken as a stationary potential energy landscape, in which simulated endo-lysosomes move along the Markov Chain given by the Metropolis criterion (Fig. S5A). In other words, particles resembling endo-lysosomes ‘move’ to regions with lowest values of the EDT map, corresponding to energy minima, i.e., regions of attraction to mitochondria until they reach their thermodynamic equilibrium distribution in the statistical sense. (Fig. 4A, most right panel and Fig. 4B). Accumulation of particles mimicking LE/LYSs around mitochondria is thereby a consequence of attraction defined by the EDT and simulates the binding process, underlying formation of MCSs between mitochondria and endo-lysosomes. The thermal energy of the simulated endo-lysosomes comes from the Boltzmann factor, and sampling is based on the Metropolis criterion^20,23^. By varying the scaling factor for the EDT map, shown in Fig. 4G, we can vary the steepness of the potential energy landscape and thereby control the extent of vesicle clustering around mitochondria. We simulated first 288 endo-lysosomes interacting with a Lennard Jones potential and being strongly attracted to mitochondria. Under those conditions, all vesicles cluster around mitochondria to an extent, which exceeds the experimentally observed clustering (compare Fig. 4C and D, and Fig. 4E and F). This is seen in the simulated images and reflected in the narrower distribution of distances to mitochondria for simulated endo-lysosomes compared to the experimental ones (Fig. 4I blue and red bars and curves). By including a second population of LE/LYSs which interacts more weakly with mitochondria and including an interaction term to the nucleus for both populations (the Morse Potential), we can simulate a distance distribution between endo-lysosomes and mitochondria, which closely resembles the experimental one of LE/LYSs containing Alexa546-NPC2 in the same cell (see simulation snapshots in Fig. 4H and analysis in Fig. 4I red and green curve). This result confirms our statistical analysis of the experimental data, which suggests that at least two subpopulations of endo-lysosomes containing NPC2 exist in fibroblasts; one which interacts strongly and one which interacts weakly with mitochondria. Note, that the system energy, which is in arbitrary units due to the definition of the energy landscape and the chosen scaling factors, equilibrates already during the first 100 MCMC steps (Fig. S5B). Also, the initial positional order of simulated particles is rapidly lost, ensuring that the start configuration has no impact on the simulation results (Fig. S6). Together, this ensures, that we indeed sample from an equilibrium distribution (only the last 10 simulation snapshots were converted into images and considered for analysis). In summary, by Monte Carlo simulation of physical encounters of endo-lysosomes with mitochondria, we can reconcile the experimentally observed distribution of endo-lysosome/mitochondria distances.

**Figure 5:**
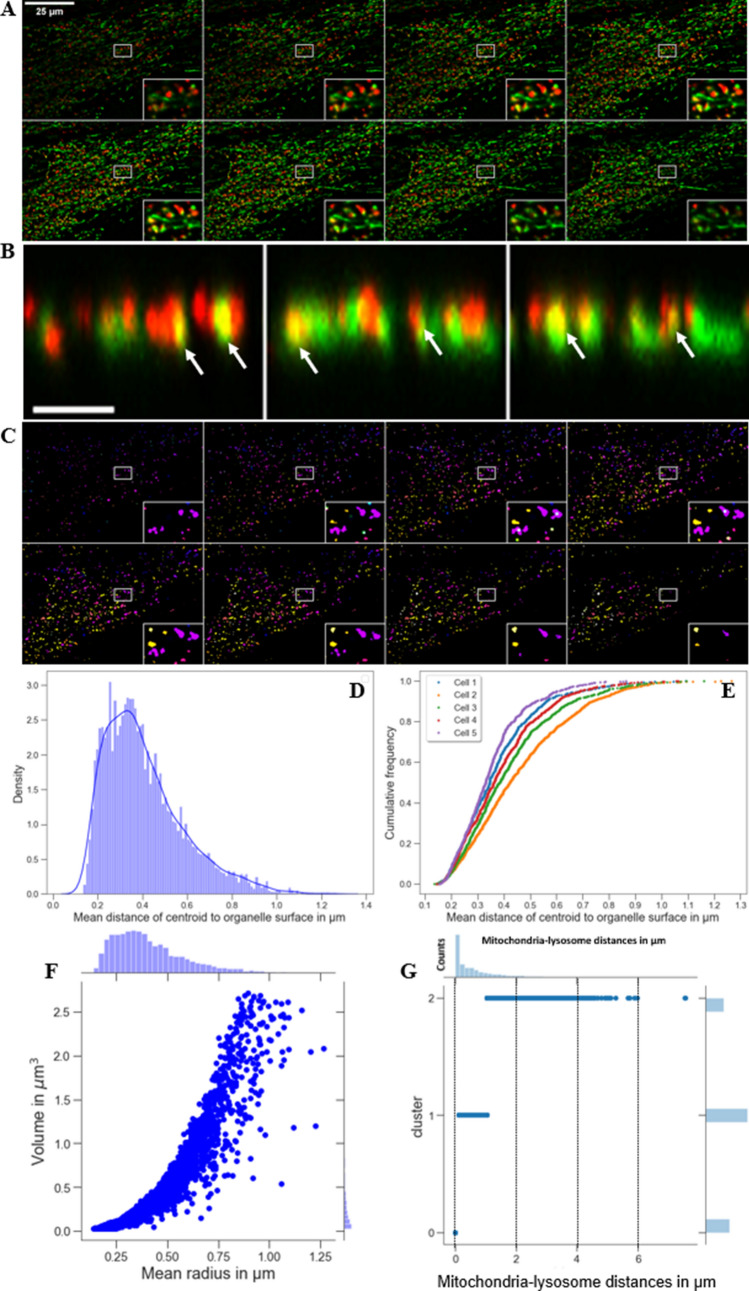
Centroid-based 3D quantification of distances between LE/LYSs containing NPC2 and mitochondria. NPC2−/− fibroblasts were incubated with 100 nM Alexa546-NPC2 for 72 h in LPDS medium and labeled for 30 min with MitoTracker Green before imaging in three dimensions at a spinning disk confocal microscope. Selected frames (**A**) and sum projections of xz-views along the optical axis (**B**) of color overlays of both channels are shown, revealing the close apposition of LE/LYSs containing Alexa546-NPC2 and of mitochondria containing MitoTracker Green (arrows in **B**). **C**, endo-lysosomes were segmented and are shown as color-coded entities together with their centroid positions for the same field as shown in A. **D**, from the 3D volume of segmented LE/LYSs the mean distance of each centroid position to the surface was calculated and plotted as histogram together with a kernel density estimation of the distance distribution. **E**, cumulative histograms of the same distances, resembling the mean radii of endo-lysosomes analyzed for each cell separately. **F**, correlation plot of these mean radii against the measured volume of the LE/LYSs. **G**, cluster analysis of the pooled histogram of distances between centroids of endo-lysosomes and mitochondria for all five cells, determined using a Gaussian mixture model as in Fig. 3F. The x-axis shows the mitochondria-lysosome distance in µm. The y-axis of the main plot shows the three identified cluster populations, while that of the upper histogram shows the number of counts. See text for further details. Bar, 20 µm in panel A and 3 µm in panel B.

Some of the LE/LYSs appearing as being in direct contact with mitochondria in 2D images could locate in fact above or below a studied mitochondrion. To account for this possibility, we extended the above analysis to three dimensions using two-color confocal z-stacks of double labeled cells (Fig. 5A). In deconvolved confocal sections, endo-lysosomes co-localized extensively with mitochondria along the optical axis (Fig. 5B). Since the same endo-lysosome is found in several planes along the optical axis, a simple peak-finding algorithm would overcount the number of LE/LYSs. We therefore used a 3D object segmentation by which endo-lysosomes containing Alexa546-NPC2 were identified as individual objects (Fig. 5C; see Materials and Methods). This allowed us to determine all positions of LE/LYSs based on the three coordinates of their centroids. The mean distance of the centroid to the organelle surface is a measure of the average organelle radius, when approximating each LE/LYSs as a sphere. The distribution of these radii of all 3D endo-lysosomes pooled from five cells was rather broad with a mean radius of 0.398 ± 0.165 µm (Fig. 5D; N = 4615). The cumulative histogram of this mean radius calculated for each of the five cells reveals some cell-to-cell heterogeneity but also shows that between 20–60% of all LE/LYSs have a radius of at least 0.4 µm corresponding to an approximate diameter of 0.8 µm (Fig. 5D and E). The size heterogeneity of endo-lysosomes containing Alexa546-NPC2 was also reflected in a rather broad distribution of organelle volumes (Fig. 5F).

To find 3D distances to mitochondria, the centroid position of all LE/LYSs pooled from five different cells was mapped onto a 3D version of the EDT calculated from the corresponding z-stack of the MitoTracker images. Note that the EDT has in three dimensions to account for the asymmetric voxel size, for which we used another program than in 2D (see Materials and Methods for details). Also, we kept the physical dimensions of the mitochondria in the 3D analysis, as obtained after thresholding and binarization, i.e. their factual volume. Accordingly, the smallest possible distance between endo-lysosomes and mitochondria in our 3D analysis is 0.0 µm, exactly when centroid positions of LE/LYSs locate to the binarized volume of mitochondria. Applying a Gaussian mixture model to the distance distribution between LE/LYSs and mitochondria, we found again three populations; the first population are those endo-lysosomes, whose centroid positions falls within the volume determined from the binarized mitochondria volume. This population comprises 28.36% of all endo-lysosomes (Fig. 5 G, ‘cluster 0’). The second population of LE/LYSs locates on average 0.439 ± 0.266 µm from the next mitochondrion and comprises 50.0% of all endo-lysosomes containing fluorescent NPC2 (Fig. 5G, ‘cluster 1’). The third population of endo-lysosomes is broadly distributed and on average 1.912 ± 0.879 µm from the next mitochondrion (Fig. 5G, ‘cluster 2’). While the first population of LE/LYSs likely forms MCSs to mitochondria according to our distance definition, a portion of the second population will eventually do that as well. This can be inferred from a comparison of the mean distance of this population to mitochondria with the mean radius of endo-lysosomes (compare Fig. 5D, E and G). We conclude from our combined experimental and computational analysis, that at least 30% of all LE/LYSs containing Alexa546-NPC2 are close enough to mitochondria to establish MCSs to these organelles.

**Figure 6:**
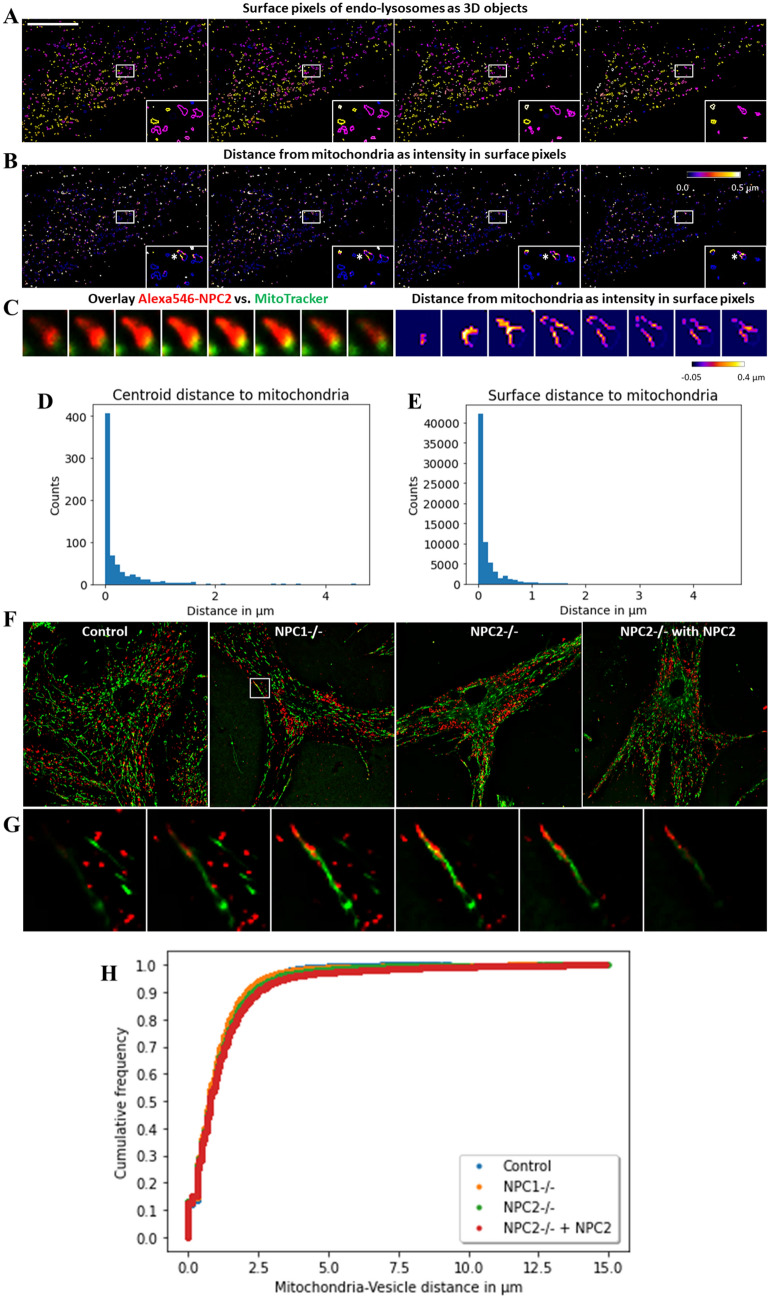
Surface-based 3D quantification of distances between LE/LYSs and mitochondria and comparison between different fibroblast types. **A**, NPC2−/− fibroblasts were incubated with 100 nM Alexa546-NPC2 for 72 h in LPDS medium and labeled for 30 min with MitoTracker Green before imaging in three dimensions at a spinning disk confocal microscope. NPC2-containing endo-lysosomes were segmented in 3D, and surface pixels of identified objects are shown for selected frames (**A**). Euclidian distances calculated in 3D based on the corresponding MitoTracker stacks were defined for the surface pixels of the LE/LYSs using a binary mask and are given as intensity values (**B**). A zoom of the endosome in the inset in **B** (next to the white star) is shown in the right half of **C**, while the corresponding color-overlay of the same endo-lysosome is shown in the left half of **C** (compare Fig. 5A). 3D Euclidian distances of 0 µm refer to regions of the vesicle surface, were the endo-lysosome is in contact with mitochondria. The centroid- and surface-based distance measurement gave comparable results, as inferred from the respective histograms calculated for the cell shown in **A** (**D** and **E**). **F**–**H**, control fibroblasts (F, ‘Control’), NPC1-deficient fibroblasts (F, ‘NPC1−/−’) or NPC2-deficient fibroblasts, either left untreated or treated with 200 nM bovine NPC2 for 24 h in LPDS (F, ‘NPC2−/−’ and ‘NPC2−/− with NPC2’, respectively) were loaded with 0.5 mg/ml Rh-dextran over night before labeling for 30 min with MitoTracker Green and 3D imaging at a spinning disk confocal microscope. Bar, 25 µm. G, montage of z-stack of zoomed box in NPC1−/− cells shows close contact of LE/LYSs and mitochondria. **H**, cumulative histogram of distances to mitochondria of all surface pixels of endo-lysosomes containing Rh-dextran were quantified for four cells in each condition. See text for further explanation.

The centroid-based analysis presented above does not account for the heterogeneous size and shape of endo-lysosomes and provides no information about the actual surface area of LE/LYSs in contact with mitochondria. To overcome this limitation, we developed a complementary approach which enables assessing the distance of the surface of all LE/LYSs from mitochondria in 3D. More precisely, we determine surface pixels of all endo-lysosomes segmented in 3D and map them to the 3D version of the EDT, which provides directly those pixels belonging to LE/LYSs which are in contact with mitochondria (Fig. 6A-C). The results of this analysis compare favorably with the centroid based 3D analysis of MCSs, as shown in the histograms of centroid distances versus surface pixel distances (Fig. 6D and E). Thus, endo-lysosomes which form contacts to mitochondria based on the centroid-distance criterion, also have a certain fraction of their surface in direct contact with mitochondria. We applied the surface-based analysis to control fibroblasts, to NPC1- and NPC2-deficient fibroblasts as well as to NPC2-deficient cells additionally treated with unlabeled NPC2 protein (Fig. 6F–H). Those cells were labeled with Mitotracker Green and additionally with rhodamine-dextran (Rh-dextran), a fluid phase endocytosis marker, which accumulates in LE/LYSs^14^. By visual inspection of the images, we found examples of close contact between endo-lysosomes and mitochondria in all cell types and conditions, as exemplified in Fig. 6G for NPC1-deficient cells. Using the surface-based 3D distance analysis we found that about 10% of the surface area of all dextran containing endo-lysosomes is in direct contact with mitochondria (i.e. distance to mitochondria equals 0 µm), irrespective of the cell type studied (Fig. 6H). These results lead to two important conclusions; 1) MCSs between endo-lysosomes and mitochondria are not limited to NPC2-containing LE/LYSs and 2) NPC1 and NPC2 are not directly involved in contact site formation. In an additional co-localization analysis, we found that about 45% of all LE/LYSs containing Rh-dextran also contain a green fluorescence labeled NPC2, Alexa488-NPC2, and that both vesicle populations cluster at a spatial scale of 0.1 to 0.4 µm (Fig. S7). Colocalization of Alexa488-NPC2 with Alexa647-tagged transferrin (Alexa647-Tf), a marker for early and recycling endosomes was also observed but was much lower than that with Rh-dextran (Fig. S7G), confirming our previous analysis^26^. We conclude that NPC2 resides primarily in a subpopulation of endo-lysosomes, and that LE/LYSs, even with different cargo, localize near each other in the cell on a spatial scale of less than 1 µm.

### Frequency and dynamics of contacts between NPC2-positive LE/LYSs and mitochondria

**Figure 7:**
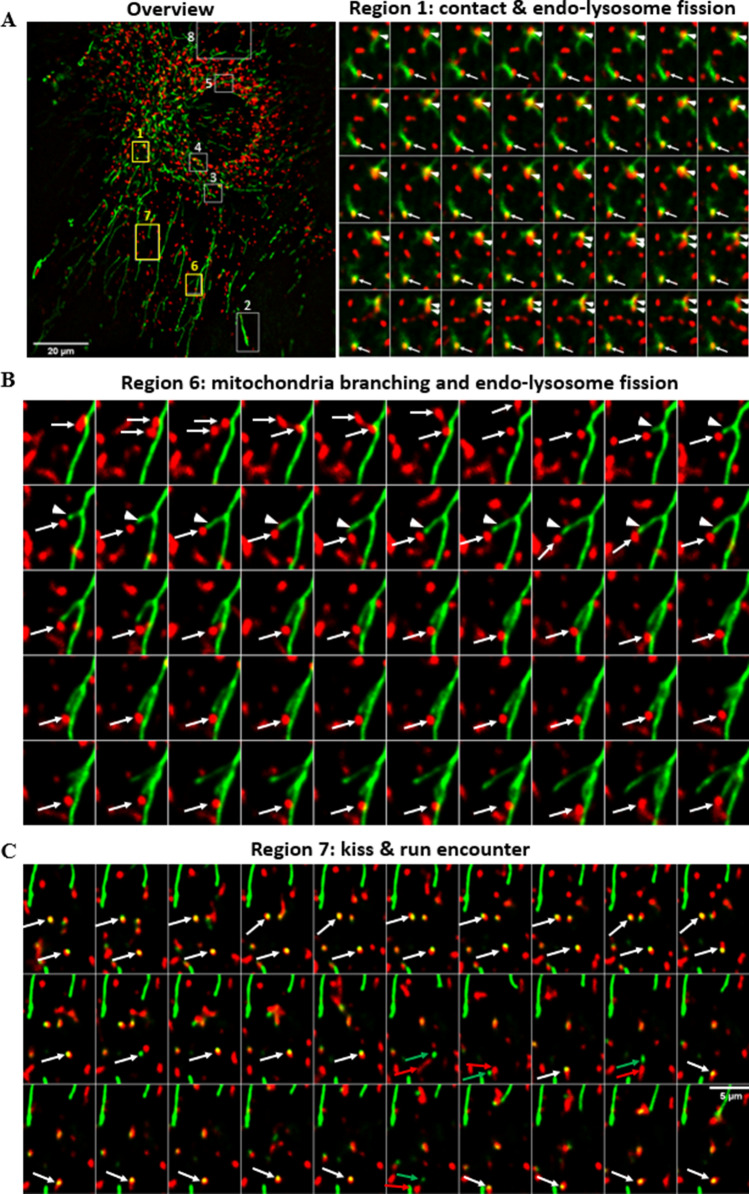
Dynamics of contact formation between endo-lysosomes and mitochondria. NPC2−/− fibroblasts were incubated with 100 nM Alexa546-NPC2 for 72 h and labeled for 30 min with MitoTracker Green before imaging at a spinning disk confocal microscope. Images were acquired every 1 s for 3 min, while cells were kept at 37 °C, 5% CO_2_. A, first frame of whole field with zoomed regions indicated. Only regions 1, 6 and 7 (yellow boxes) are shown in the right panel of **A**, in **B** and **C**, respectively. These and the other boxes are shown as Supplemental videos. Bar, 20 µm. The montage of region 1 shows every second image (i.e. every 2 s), while the montages of region 6 and 7 show every third image (i.e. every 3 s).

To obtain insight into the dynamics of MCSs between endo-lysosomes and mitochondria, we carried out time-lapse experiments, in which we focused on NPC2-deficient cells loaded with red fluorescent Alexa546-NPC2 and co-stained mitochondria with MitoTracker Green. Images were taken every 1 s on a temperature- and focus-controlled spinning disk confocal microscope. We observed that LE/LYSs can undergo fission while being in contact with mitochondria (Fig. 7A and Supplemental video 1). Fusion and fission of mitochondria were repeatedly observed (Supplemental video 2 and 3). Some endo-lysosomes almost slide along mitochondria before eventually split into two, suggesting that endosome fission is tightly coupled to mitochondrial dynamics, similar as previously shown for endosome-ER contacts (Supplemental video 4 and 5)^27^. A mitochondrion containing an endo-lysosome at its tip underwent branching, suggesting that both organelles were pulled by motor proteins along the same cytoskeleton track (Fig. 7B and Supplemental video 6). We also observed frequent transient encounters of spherical mitochondria with LE/LYSs containing NPC2 in a ‘kiss-and-run’ fashion, especially with punctate-shaped mitochondria (Fig. 7C and Supplemental video 7 and 8). Such small spherical mitochondria could resemble vesicles, which have been shown to bud from elongated mitochondria under metabolic stress conditions to exchange cargo with endo-lysosomes and peroxisomes^28^. Our time-lapse experiments demonstrate that interactions of endo-lysosomes with mitochondria can be highly dynamic and that fusion and fission of both organelles can be coupled to each other. On the other hand, many LE/LYSs in contact with elongated and branched mitochondria moved very little and kept the contact for tens of seconds (e.g. Supplemental video 9). Such long-term concerted motion is indicating some form of interaction between LE/LYSs and these mitochondria.

**Figure 8:**
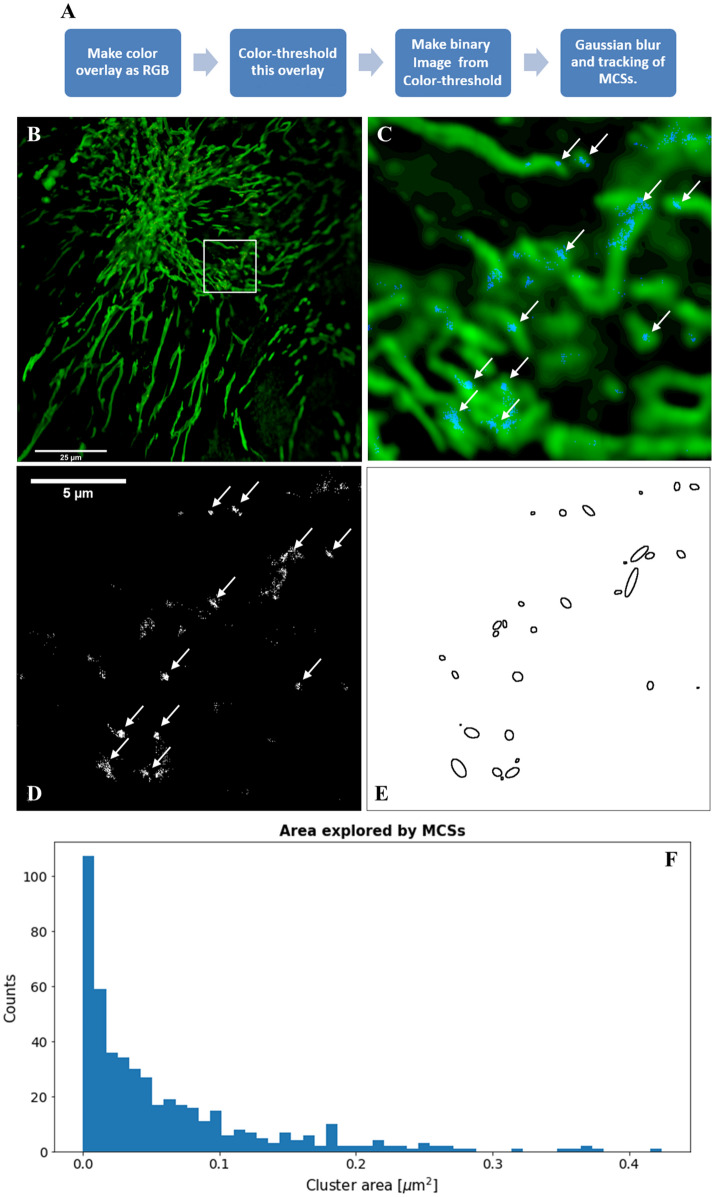
Localization microscopy reveals dynamic clusters of membrane contact sites. Workflow of the analysis is shown in **A**. Positions of MCSs identified using the segmentation procedure shown in panel A were localized using a 2D Gaussian fit and projected onto the sum projection image of mitochondria from a 3-min video recording. **B**, overview with mitochondria in green and positions of MCSs in blue. Bar, 25 µm. The 18 × 18 µm white box is enlarged in **C**, such that the blue positions of MCSs appear as small clusters on top of green mitochondria. **D**, map of identified positions of MCSs from whole video sequence showing dynamic clusters of all projected contact site locations. **E**, ellipses indicate dynamic clusters identified as individual objects for size analysis shown as histogram in panel **F** for a total of four analyzed cells.

To quantify the dynamics of MCSs between LE/LYSs and mitochondria in living cells, we devised a method for the reliable and automated quantification of MCSs in time-lapse sequences of living cells (Fig. 8A). In this method, we first applied a color-threshold to merged RGB time-lapse stacks of both channels, thereby identifying overlapping pixels in each frame (Fig. S8A-C, orange and blue spots). In a second step, these binary regions were classified as objects in a new time-lapse stack allowing for comparing their movement along the underlying mitochondrial structure. Using a time-lapse color stamper, which color codes the position of imaged features from live-cell video sequences, one can visualize the identified MCSs and finds that some of them move back and forth along mitochondria (Fig. S8D, E and F), while others are almost immobile (Fig. S8G and H). To assess this observation quantitatively, we localized all identified MCSs throughout a 3-min time lapse recording by applying a 2D Gaussian fitting routine and projected their positions from different time points onto one image map, similar as done in single molecule localization microscopy^29^. Overlaying this position map onto a sum projection of the time-lapse sequence of MitoTracker from double-labeled cells, we find that many MCSs stay confined in very distinct clusters (Fig. 8B-D). While mitochondria, of course, contribute to the dynamics of MCSs, as they are part of the segmented structure, their overall dynamics is rather limited since mitochondria can be well-discerned from the sum projection of the time-lapse sequence (Fig. 8B and C). Analyzing the size distribution of such dynamic clusters reveals that most of them cover an area smaller than 0.1 µm^2^ (Fig. 8E and F). Thus, many MCSs are rather static, and the extent of confinement suggests a strong interaction between the involved endo-lysosomes and mitochondria. Having segmented image regions of overlap between mitochondria and LE/LYSs, we can track their position over time and analyze the trajectories. From all trajectories of at least 10 s length in a given cell, we calculate the mean squared displacement (MSD) and from that the diffusion properties of MCSs. We obtained 388 trajectories of MCSs compared to 1794 trajectories of endo-lysosomes containing Alexa546-NPC2. Similar values were found for three other cells (not shown), suggesting that about 20–25% of all LE/LYSs containing fluorescent NPC2 establish MCSs to mitochondria.

**Figure 9:**
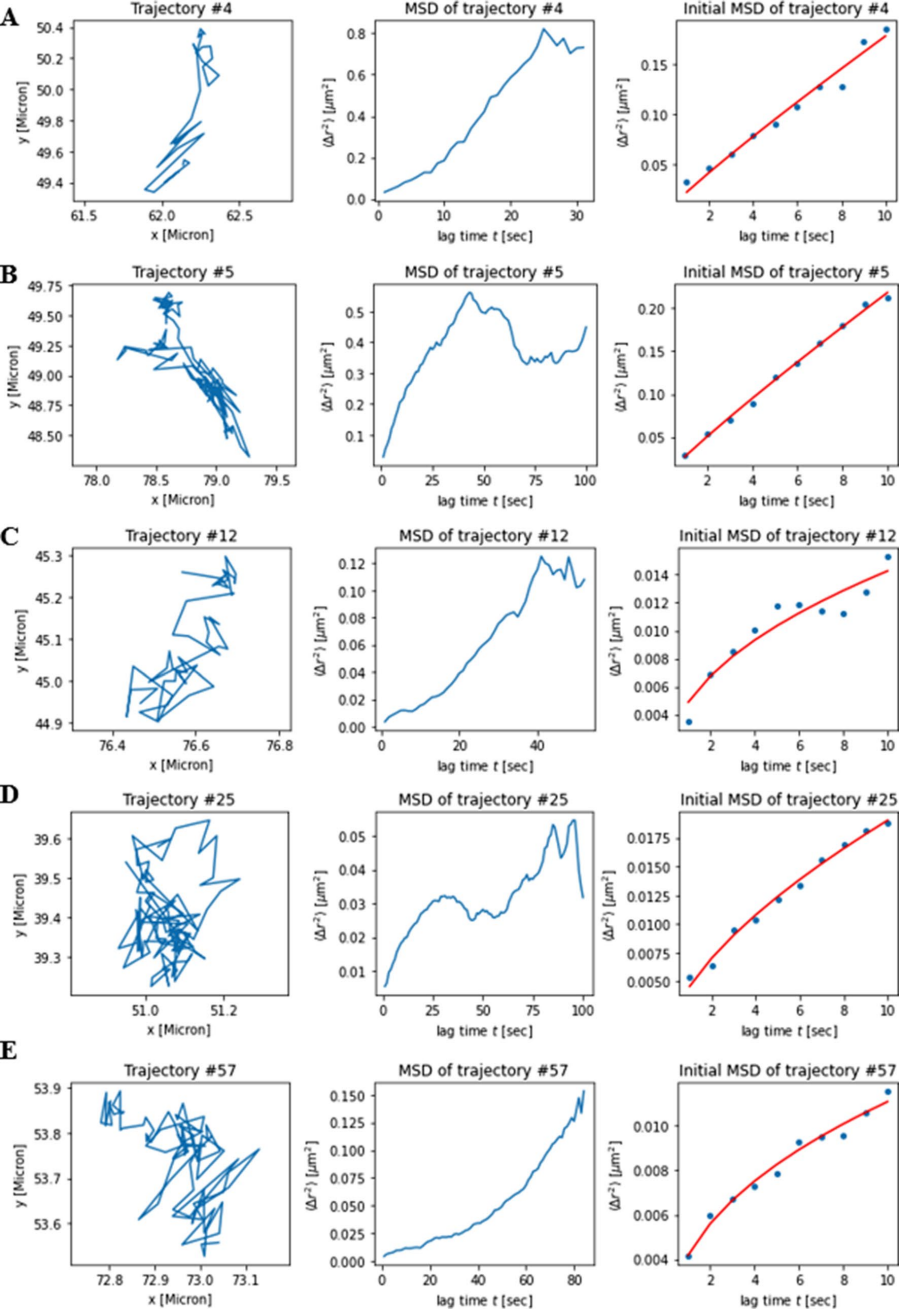
Selected trajectories of dynamic membrane contact sites and their analysis. MCSs identified in a single cell (‘cell1’, compare Fig. 7–8) were analyzed by single particle tracking revealing their trajectories (left panels), from which the time-averaged mean square displacement (MSD) was calculated (middle panels). The MSDs for the first 10 s were fit to an anomalous diffusion model, according to Eq. (1) in Materials and Methods (right panels; blue dots = data, red lines = fit). See text for further explanation.

**Figure 10:**
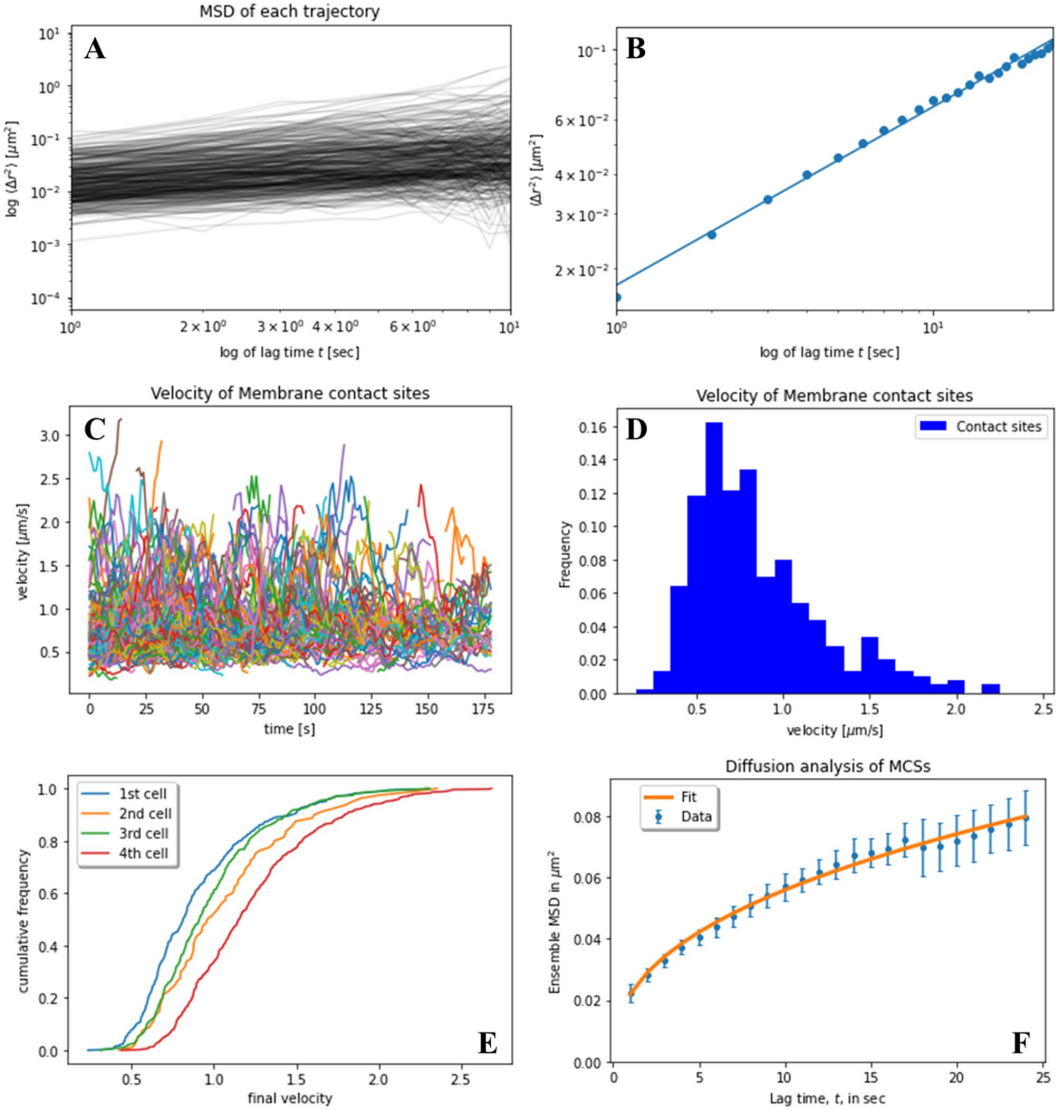
Statistical analysis of the dynamics of MCSs of entire cells, **A**, the first ten seconds of time averaged MSDs for all trajectories of a given cell (‘cell1’, compare Figs. 7, 8, 9) are shown in log–log space. **B**, the ensemble-averaged MSD for the same cell is shown in panel **B** together with a fit to an anomalous diffusion model, Eq. (1) in Materials and Methods. **C**, **D**, instantaneous velocities (**C**) and histogram of velocities (**D**) of all MCSs tracked for at least 10 s throughout the entire cell 1. Cumulative distribution of instantaneous velocities is shown for each of the four analyzed cells in panel **E**. The MSD was calculated as ensemble-average for each cells and averaged for all cells, as shown in panel **F**. Data is shown as mean + /− SEM of n = 4 cells and was fitted with an anomalous diffusion model according to Eq. (1) in Materials and Methods. See Fig. S9 for individual fits to MSDs in log–log space for cell 2 to 4, together with corresponding velocity histograms.

Examples of trajectories and corresponding MSDs are shown in Fig. 9. Many MCSs could be tracked for 30–100 s, showing that the 10-s cut off, we used for tracking is indeed a lower limit. Durations of MCSs between endo-lysosomes and mitochondria for up to 3 min have been observed previously, suggesting that our observation is not unique to NPC2-containing LE/LYSs^30^. By fitting an anomalous diffusion model to the MSDs of such MCSs, we found evidence for slow sub-diffusive motion on short times (right panels in Fig. 9). On longer time scales, some MCSs show a transition to upward curved MSDs (Fig. 9A, C and E, middle panels), while for other trajectories the MSDs reach plateau values for long times (Fig. 9B and D, middle panels). Upward curved MSDs for long times is characteristic for diffusion coupled to active transport (flow) of organelles along microtubules and other filaments, the latter depending quadratically on time^31^. Such dynamic behavior has been observed for both, LE/LYSs and for mitochondria, since both can be transported actively in cells^21,32^. To determine, whether subdiffusion on short time scales is a general trend for MCSs between LE/LYSs and mitochondria, we analyzed time-averaged MSDs of all 388 identified trajectories for this cell and compared it to the ensemble averaged MSD (Fig. 10A and B). The time-averaged MSDs differ significantly from each other, suggesting that the dynamics of MCSs is rather heterogeneous^33^. The ensemble-averaged MSD could be well-described by an anomalous diffusion model, and the same holds for three additional cells analyzed in the same way (Fig. 10B, S9). The parameters estimated for each cell were averaged (n = 4) giving + /- standard error of the mean: D = 0.0055 ± 0.0005 µm^2^/s, α = 0.4110 ± 0.0505). In all cases, we found a left-skewed distribution of instantaneous velocities with a peak around 0.6 µm/s and maximal values up to 2.3 µm/s (Fig. 10C,D and S9). About half of all tracked MCSs had step-to-step velocities between 0.5 and 1.0 µm/s, as inferred from the cumulative velocity distribution (Fig. 10E). The mean of the ensemble averaged MSD from all four cells could also be well-described by the anomalous diffusion model (Fig. 10F). Together, we conclude that LE/LYSs containing Alexa546-NPC2 can interact with mitochondria for 10 s and longer during which the contact areas undergo slow sub-diffusive motion. Such long-term contact formation could pave the way for cholesterol transfer from LE/LYSs to mitochondria.

## Discussion

Mitochondria are not only the central organelles for carrying out oxidative metabolism, but also the site of production of steroid hormones and oxysterols from the precursor cholesterol. Exchange of metabolites, ions and lipids between mitochondria and other organelles can take place via MCSs, but few tools are available to study such MCSs quantitatively in living cells. This contrasts with quantitative analysis of mitochondrial morphology, for which several image analysis toolboxes have been developed in recent years, e.g.^17,34–37^. We present two novel approaches to quantify the spatiotemporal relationship between endo-lysosomes and mitochondria from live-cell imaging data. Here, we devise a novel analysis strategy based on object segmentation, distance transforms and probabilistic modeling to automatically assess distances between mitochondria and endo-lysosomes. We developed two methods based on this approach; (1) centroid-based and (2) surface-based distance measurement between both organelles types. The first method measures the distance between the centroid positions of all LE/LYSs to their nearest mitochondria and thereby allows for identifying a subpopulation of endo-lysosomes forming close contact to mitochondria. Using 3D confocal microscopy, we demonstrate that such contacts between both organelle types are not due to 2D projections of the cellular volume. The second method for distance measurements quantifies the distance of each surface pixel of the individual endo-lysosomes to nearby mitochondria in 3D. This method provides information of the surface fraction of all LE/LYSs being in contact with mitochondria. Both methods have been automatized and are available as Macros to ImageJ from the authors. We show that both methods provide complementary information about the extent of contact site formation. To study the dynamics of MCSs between LE/LYSs and mitochondria in living cells, we employ color segmentation combined with object tracking to identify a subpopulation of all MCSs, which remains stable for at least 10 and up to 100 s. Such long-lasting MCSs move slowly and in strongly confined areas, well described by a model for anomalous sub-diffusion. Whether the subdiffusive motion of MCSs is due to spatial confinement of bound endo-lysosomes in a constricted space of the cytoplasm and/or the consequence of viscoelastic effects on the mitochondria-vesicle complexes remains to be shown in future analyses.

To assess mitochondrial ultrastructure and resolve MCSs at the nanoscale, we employ X-ray microscopy. SXT provides three-dimensional information with isotropic resolution ≤ 50 nm, and since its contrast is based on linear absorption of X-rays, the extent of ‘darkness’ in reconstructed transmission images is a quantitative measure of X-ray absorption by cellular structures^38,39^. By SXT we can resolve the internal structure of mitochondria, revealing the organization of cristae and detect areas of close apposition of vesicular organelles, resembling LE/LYSs. The complex mitochondrial ultrastructure, we observe by SXT in human fibroblasts is in accordance with recent X-ray and electron microscopy studies of mitochondria in muscle cells^39,40^. It supports the potential of these methods in delineating mitochondrial structure and morphology in human diseases.

Mitochondria have been proposed to receive cholesterol either from the PM or from LE/LYSs and additionally from lipid droplets^2^. Evidence for such transport pathways comes from biochemical experiments on purified mitochondria^41,42^. Alternatively, measurements on formation of pregnenolone, the first step in steroid hormone synthesis from cholesterol taking place in mitochondria^9,15^ or analysis of synthesis of 27-hydroxycholesterol, an oxysterol formed from cholesterol in mitochondria^43^ can report about cholesterol transport to mitochondria. Such studies have shown, that NPC2 is needed to deliver cholesterol from endosomes to mitochondria, while NPC1 is not essential^9,15^. Supporting this model, Karten and co-workers showed that NPC2 mutant proteins which can transfer sterol between membranes in vitro but cannot bind to NPC1, can rescue pregnenolone production in NPC2 deficient cells^9,44^. Mitochondria from NPC1-deficient cells were even found to be enriched in cholesterol which was accompanied by various metabolic dysfunctions^44,45^. Sterol export from LE/LYSs containing NPC2 to mitochondria could proceed via the sterol transporter MLN64 (also called StARD3), which locates to the same endo-lysosomes as the NPC proteins, and whose expression is upregulated in NPC1 deficient cells^46–48^. To study sterol transport, we used the fluorescent cholesterol analog CTL, which resembles cholesterol closely and accumulates in endo-lysosomes of NPC1 (Fig. S10) and NPC2 deficient cells^13^. By live-cell imaging of CTL, we observe sterol transport to mitochondria in dependence of NPC2 but independent of NPC1, strongly supporting such biochemical studies. Involvement of MLN64 as an acceptor for sterol delivered by NPC2 in the endo-lysosomal membrane is further supported by the observation, that ectopic expression of MLN64 in NPC2-deficient cells did not rescue the cholesterol storage phenotype, suggesting that both proteins operate in the same pathway^49^. Based on these and our results, we suggest that the MCSs we observed between LE/LYSs containing NPC2 and mitochondria could serve the function of sterol transfer between both organelles. Since in our labeling procedure, a lot of the CTL resides in the PM, we suggest that CTL traffics from the PM first to LE/LYSs containing NPC2 and from there to mitochondria in dependence of NPC2 function. Supporting such a model we have shown recently, that cycling of sterol between PM and LE/LYSs depends on NPC2^14^. While we believe that this is the most likely scenario, our study does not rule out that other sterol trafficking pathways contribute to cholesterol transport to mitochondria, like transport from droplets or via de novo cholesterol synthesis. Also, a recent study found NPC2 in a mitochondria-associated population of autophagosomes and implicated NPC2 in Toll-like receptor mediated activation of autophagy, in adipocytes^50^. Since autophagosomes are formed from LE/LYSs and control mitochondria turnover^51^, an additional role of MCSs between mitochondria and endo-lysosomes containing NPC2 could be to control autophagy and mitophagy.

While NPC2 is important for sterol transport from LE/LYSs to mitochondria, we found that neither NPC1 nor NPC2 is involved in establishing contact sites between both organelle types (Fig. 6). Thus, other proteins must provide the molecular machinery for organelle tethering, such as specific binding partners for the rab GTPase rab7^30^. Constitutively active rab7 increased the number and duration of contacts, while the rab7 GTPase activating protein TBC1D15 terminated contacts between endo-lysosomes and mitochondria in HeLa cells^30^. Interestingly, NPC1 has been implicated in MCSs between the ER and endo-lysosomes, where rab7-NPC1 interactions via the C18orf8-Mon1-Ccz1 guanidine exchange factor were shown to control lysosomal cholesterol export^52^. In light of these and our results, involvement of NPC1 in membrane tethering seems to be organelle and eventually cell-type specific, and an interesting question would be to identify the rab7 interaction partners for contact formation to mitochondria. Some of the discrepancy concerning the involvement of NPC1 in formation of inter-organelle contact sites could also originate in different imaging modalities and employed image quantification protocols. The automated cell-wide image analysis methods, we provide here will allow one to address this and other questions in a user-unbiased manner in future studies.

Apart from cholesterol transport, MCSs could play a role in ion homeostasis between endo-lysosomes and mitochondria. For example, delivery of iron from internalized transferrin into mitochondria has been shown to require transient tight contacts to endosomes^53,54^. Similarly, MCSs between LE/LYSs and mitochondria regulate mitochondrial calcium dynamics in a process involving the lysosomal cation channel TRPML1^55^. TRPML1 has been shown to be less active in lysosomal storage disorders including NPC1 deficiency and calcium signaling has been reported to be disturbed in NPC1 deficient cells^56,57^. An attractive hypothesis is, therefore, that NPC2 also plays a role in calcium signaling between endo-lysosomes and mitochondria, either directly or indirectly, via its function as cholesterol carrier protein delivering sterols to NPC1 for export from the LE/LYSs. This hypothesis can be tested in future studies using the tools developed here.

## Material and methods

### Reagents

Fetal bovine serum (FBS) and DMEM were from GIBCO BRL (Life Technologies, Paisley, Scotland). Other chemicals including human lipoprotein depleted serum (LPDS), EMEM (51417C) and DHE were from SIGMA Chemical (St. Louis, MO). MitoTracker Green, rhodamine-dextran (Rh-dextran, 70 kD) and succinimidyl esters of Alexa488, Alexa546 and Alexa647 were purchased from Invitrogen/Molecular Probes (Inc. USA). NPC2 was purified from bovine milk and conjugated with an succimidyl ester of Alexa488 (emission in green) or of Alexa546 dye (emission in red) as described previously^21^. Transferrin was iron-loaded and subsequently labelled with the Alexa647 dye as described previously^14^. Cholestatrienol (CTL) was synthesized as described^58^.

### Cell culture

Human skin fibroblast from healthy male donor (Coriell Institute #GM08680 (referred to as control)) and from NPC2 patients (Coriell Institute #GM18455) were purchased from Coriell Institute for Medical Research (NJ, USA). They were cultured in T25 culture flasks, at 37^o^ C in an atmosphere of 5% CO_2_ in complete DMEM culture medium supplemented with 1% glutamax, 1% Penicillin–Streptomycin and 10% or 20% FBS respectively. Primary fibroblasts from NPC1 patients (Coriell Institute #GM03123) were cultured under same conditions, in 15% FBS in EMEM supplemented with 1% Penicillin–Streptomycin. Cells were checked daily and split with trypsin when a confluency of 90% were reached. Prior to fluorescent microscopy, cells were placed on 35 mm microscope dishes with glass bottom (P35G-1.5–50-C, MatTek) (coated with poly-D-lysine for NPC2 diseased cells) and allowed to settle for 48–72 h in their culture medium. All live cell imaging was carried out in M1 buffer containing 150 mM NaCl, 5 mM KCl, 1 mM CaCl_2_, 1 mM MgCl_2_, 5 mM glucose and 20 mM HEPES (pH 7.4) as described^59^. All experiments on cells were carried out in accordance with ethical guidelines and safety regulations defined by the provider Coriell Cell Repositories (www.coriell.org) and the University of Southern Denmark.

### Labeling of cells

For labeling with sterols, a CTL bovine serum albumin complex (CTL/BSA) was made by mixing 100 mg BSA in 2 mL PBS with 200 μM CTL from an ethanol stock. The mixture was vortexed for 5 min and left to attain equilibrium for 30–60 min and stored under N_2_ at 4^o^ C. Cells were loaded with 200 μL CTL/BSA in 1.8 mL LPDS medium for 48 h and subsequently allowed 24 h chase time in LPDS medium with or without 200 nM NPC protein. Prior to wide field microscopy, the cells were loaded with MitoTracker green as described below. A DHE/BSA complex was made as described above for CTL/BSA^26^. For co-localization experiments of endosome-lysosome markers, NPC2-deficient fibroblasts were first labeled with DHE/BSA in medium containing LPDS for 48 h (to identify the cell borders), washed and incubated in medium with LPDS and 100 nM Alexa488-NPC2 and 0.5 mg/ml Rh-dextran for another 24 h followed by a 30-min incubation in medium with LPDS and 5 µg/ml Alexa647-Tf. MitoTracker Green FM was dissolved in dimethylsulfoxide to a final concentration of 1 mM and diluted to a final concentration of 20 μM in PBS. NPC2 diseased fibroblasts grown on poly-D-lysine coated microscope dishes were incubated with 100 nM Alexa546-NPC2 for 72 h in LPDS medium. Prior to imaging, the cells pre-loaded with Alexa546-NPC2 or with Rh-dextran were loaded with 200 nM MitoTracker in M1 buffer for 30 min at 37^o^ C. In separate experiments, control, NPC1 deficient or NPC2 deficient fibroblasts (the latter prior to or after incubation with 200 nM bovine NPC2 for 24 h) were loaded with 0.5 mg/ml Rh-dextran over night before labeling for 30 min with MitoTracker Green and 3D imaging at a spinning disk confocal microscope.

### Fluorescence microscopy

Wide field epifluorescence microscopy was carried out on a Leica DMIRBE microscope with a 63 × 1.4 NA oil immersion objective (Leica Lasertechnik GmbH) controlled by a Lambda SC smart shutter (Sutter Instrument Company). Images were acquired with an Andor Ixon^EM^ blue EMCCD camera driven by the Solis software. CTL was imaged in the UV using a specially designed filter cube obtained from Chroma Technology Corp. with 335-nm (20-nm bandpass) excitation filter, 365-nm dichromatic mirror and 405-nm (40-nm bandpass) emission filter. MitoTracker Green and Alexa488-NPC2 were imaged using a standard fluorescein filter set [470-nm, (20-nm bandpass) excitation filter, 510-nm longpass dichromatic filter and 537-nm (23-nm bandpass) emission filter]. Rh-dextran was imaged using a rhodamine-filter cube [535-nm, (50-nm bandpass) excitation filter, 565-nm longpass dichromatic filter and 610-nm (75-nm bandpass) emission filter], while Alexa647-Tf was imaged using an infrared filter cube [620-nm, (20-nm bandpass) excitation filter, 660-nm dichromatic filter and 700-nm (75-nm bandpass) emission filter]. Spinning disk confocal microscopy was carried out on a Nikon Ti-E spinning disc microscope with a 60 × NA 1.4 oil objective, a Yokagawa CSU-X1 spinning disk and equipped with an Okolab microscope stage incubator to maintain the temperature at 37 °C and 5% CO_2_. The laser lines 491 nm and 561 nm were used for MitoTracker green and Alexa556-NPC2 or Rh-dextran, respectively. An electron-multiplying CCD camera (Andor iXon EMCCD DU-885, 1004 × 1002 pixel, 8 × 8 μm) was used to acquire the images on that instrument.

### Image analysis of fluorescence microscopy data

All image analysis was done using ImageJ (http://rsb.info.nih.gov/ij), and data was further analyzed using self-developed Jupyter notebooks (https://jupyter.org/) and R-scripts (https://www.r-project.org/; available upon request).

#### Image preprocessing

Multichannel fluorescence images were routinely deconvolved using a theoretical point spread function of the appropriate wavelength, numerical aperture, refractive index and pixel spacing and by applying 30 iterations of the Richardson-Lucy algorithm including background correction in the ImageJ plugin DeconvolutionLab^60^. Fluorescence of CTL was separated from cellular autofluorescence in the UV channel by acquiring bleach stacks of labeled cells, followed by subtracting the last image resembling autofluorescence (frame 50), from the first being comprised of CTL and autofluorescence (frame 1). This procedure was validated by measuring bleaching kinetics relative to unlabeled cells using PixBleach, as described previously^61^.

#### Quantification of distances between LE/LYSs and mitochondria

To determine positions of LE/LYSs containing Alexa546-NPC2 a Macro was written which implements the FindMaxima routine in ImageJ excluding edge maxima. Identified peak positions were saved as regions of interest (ROIs). Using another Macro, the corresponding mitochondria images were first segmented using an intensity threshold to get binary images followed by skeletonization. The skeletonized images were inverted, and the Exact Euclidian Distance Transform (3D) plugin of ImageJ was called from the Macro to get an EDT map for each mitochondria image. The calculated distances were converted to µm, and the positions of all endo-lysosomes containing Alexa546-NPC2 were mapped onto the EDT. The resulting distance values of LE/LYSs from six cells (n = 3560 distances) were read-out to an ImageJ Results table from which the distribution of inter-organelle distances was determined for statistical inference of the underlying distribution. This inference consisted of (a) fitting the experimental distribution with the sum of three Gaussian functions (Fig. S4A) and (b) application of a Gaussian mixture model using the scikit learn library for Python (https://scikit-learn.org/stable); see Fig. 3). For 3D segmentation of LE/LYSs from confocal z-stacks, we used the 3D ImageJ suite developed by Dr. Thomas Boudier^62^ and available at https://imagejdocu.tudor.lu/plugin/analysis/3d_analysis/start. This plugin provides statistical measures of organelle sizes but also the surface pixels of identified objects in three dimensions. The centroids and surface pixel maps were calculated for LE/LYSs labeled either with Alexa546-NPC2 or with Rh-dextran. Using an intensity threshold, the surface pixel map was binarized in 32-bit format, background pixels were set to NaN, and this binary mask was applied to the 3D-EDT thereby allocating 3D distances to neighboring mitochondria as intensity values to the surface pixels. All pixel intensities were measured in an automatized manner in an ImageJ Macro script and further analyzed in a Jupyter notebook.

#### Segmentation and tracking of MCSs

Corresponding image stacks of fibroblasts labeled with Alexa546-NPC2 and MitoTracker Green were overlayed in an RGB color stack. Color-thresholding implemented in ImageJ was applied followed by conversion to a binary mask of segmented MCSs and blurring with a Gaussian filter with one-pixel width. Using the ImageJ plugin ThunderStorm developed for localization microscopy^63^, the coordinates of all identified MCSs were determined and mapped onto a time projection (i.e. average shifted histogram using a scaling factor of 5). The identified dynamic clusters were slightly blurred with a Gaussian filter to merge sub-resolution clusters, and their sizes were determined using binarization and particle counting in ImageJ (see Fig. 8D and E). Tracking of MCSs was carried out either with SpatTrack, a MatLab-based program developed by us^21^, or using the Python library Trackpy developed by D. Allan and co-workers^64^. Both programs implement the Crocker and Grier algorithm for particle tracking^65^, but Trackpy is Python-based and allows additionally for correction of drift which can take place during image acquisition. Time- and ensemble MSD as well as velocity distributions were calculated using Trackpy in self-developed Jupyter notebooks.

The MSD was fitted to an anomalous diffusion model of the form^26^:

1 $$ MSD\,(t) = D_{\alpha } \cdot t^{\alpha } $$

Here, *D*_α_ is the (anomalous) diffusion constant, and *α* is the anomaly parameter. For α < 1, the model describes subdiffusion, for α = 1, normal (free) diffusion and for α > 1, superdiffusion.

#### Analysis of mitochondrial length and morphology

The free available Mitochondrial Network Analysis (MiNA) toolset for Fiji was used to analyze the length and branching of mitochondria in single cells, of deconvolved images^17^.

#### Co-localization analysis of Rh-dextran and and Alexa647-Tf with Alexa488-NPC2

Deconvolved widefield images were imported into SpatTrack^21^, and particle-detection based colocalization analysis was carried out using a pixel size of 0.233 µm and a suitable tolerance for particle displacement between image acquisition. Intensity-thresholding of the corresponding DHE image allowed for identifying cell borders upon binarization. In addition to calculating the fraction of co-localized endo-lysosomes in each channel, SpatTrack allows for calculating the nearest neighbor distance between both markers. It also calculates the radial distribution function (RDF) within the cell geometry given by the mask image to identify cell borders and carries out a simulation of randomly placed vesicle populations for each channel to normalize the RDF^21^. The RDF gives thereby the spatial scale at which different endo-lysosome populations coincide relative to a random particle distribution (Fig. S7)^21^.

### Image simulations

#### MCMC simulations of endo-lysosome distributions relative to mitochondria in the cell geometry

The MCMC simulation was implemented as Macro to ImageJ. Two populations of vesicles were simulated, interacting by a Lennard Jones potential and additionally by a Morse-type potential to mimic the impact of the nucleus as recently described^20^, and illustrated in Fig. S5.

An additional contribution to the energy function came from a scaled version of the EDT-map. The strength of the interaction with mitochondria is modeled with a scaling factor *f* = 100 multiplied with the EDT map for the red particles, meaning that the potential energy contribution for the EDT map varies in this case map between 0 kcal/mol exactly at positions of mitochondria (i.e., 0 Å) to 1000 kcal/mol at the largest distance of 10 Å. Additionally an equally sized population of particles with weaker interaction to mitochondria (*f* = 10 or f = 0 for the green particles) was included. Particles were randomly displaced with distances drawn from a Gaussian distribution, which was implemented using the Box-Muller procedure as ImageJ Macro, as described^66^. Moves were accepted/rejected based on the Metropolis criterion.

### Soft X-ray tomography

#### Cell preparation

R 2/2 grids (QUANTI-FOIL, 100 Holy Carbon Films, Grids: HZB-2 Au) were tapped to objective glasses, that had been cut into appropriate pieces to fit the bottom of 12 well plates and autoclaved. Before seeding NPC2 diseased fibroblasts, the grids were coated with poly-D-lysine. The cells were allowed to grow in their culture medium to reach a confluency of approximate 70%, before they were treated with 100 nM NPC2 protein for 48 h in LPDS medium. Prior to fixation with 4% paraformaldehyde at room temperature, the cells were pulse labeled with TopFluor cholesterol (TF-chol) from a methyl-β-cyclodextrin complex (TF-chol/MCD) for 3 min and chased for 2 h in M1 media at 37 °C. The signal from TF-chol were used to localize cells before acquiring tomograms. The cells were kept in PBS until cryo-plunge freezing with liquid ethane and subsequently storage in liquid nitrogen. Before plunge freezing, a small volume of 270 nm gold beads was added to the grids to serve as fiducial markers for subsequently tomographic alignment and reconstruction.

#### Transmission X-ray microscopy

SXT was performed at beamline U41-PGM1-XM at the electron storage ring BESSY II operated by Helmholtz-Zentrum Berlin. During imaging, the plunge frozen cells on the grids were kept at liquid nitrogen temperature. The cells were imaged over a tilt range of 120–125° with 1° tilt steps on a full-field transmission X-ray microscope, with an X-ray photon energy of 510 eV and a 25 nm zone plate. The image pixel size was 9.8 nm. A light microscope incorporated in the X-ray microscope, with a Zeiss LD EC Epiplan Neofluar 100 × NA 75 DIC was used to collect the corresponding fluorescent signal^67^.

#### Image processing and analysis

The freely available software B-Soft and Tomo3D were used to align and reconstruct the SXT data, respectively^68,69^. The segmentation and subsequent rendering were performed in SuRVoS^70^.

## Supplementary Information

Supplementary Information 1.

Supplementary Video 1.

Supplementary Video 2.

Supplementary Video 3.

Supplementary Video 4.

Supplementary Video 5.

Supplementary Video 6.

Supplementary Video 7.

Supplementary Video 8.

Supplementary Video 9.

## Data Availability

Experimental data, R-scripts, ImageJ Macros and Jupyter notebooks for data analysis will be made available by the authors upon request.
