# Supplementary material for: Quantitative imaging of membrane contact sites for sterol transfer between endo-lysosomes and mitochondria in living cells
Source: Sci Rep. 2021 Apr 26;11:8927. doi: 10.1038/s41598-021-87876-7 (PMC8076251; doi:10.1038/s41598-021-87876-7)
Supplement: Supplementary file 1 — Supplementary Information 1. [file 41598_2021_87876_MOESM1_ESM.pdf]

Quantitative imaging of membrane contact sites for sterol transfer between endo-lysosomes and mitochondria in living cells

Alice Dupont Juhl[1], Christian W. Heegaard[2], Stephan Werner [3], Gerd Schneider[3], Kathiresan Krishnan[4], Douglas F. Covey[4] and Daniel Wüstner[#,1]

[1]*Department of Biochemistry and Molecular Biology, VILLUM Center for Bioanalytical Sciences, University of Southern Denmark, DK-5230 Odense M, Denmark,*
[2]*Department of Molecular Biology and Genetics, University of Aarhus, DK-8000 Aarhus C, Denmark.*
[3]*Department of X-Ray Microscopy, Helmholtz-Zentrum Berlin, Albert-Einstein-Str. 15, 12489 Berlin, Germany*
[4]*Department of Developmental Biology, Washington University, St. Louis, MO 63110, USA*

#Address correspondence to: Daniel Wüstner, Department of Biochemistry and Molecular Biology, University of Southern Denmark, Campusvej 55, DK-5230 Odense M, Denmark
Tel. +45-6550-2405, Fax +45-6550-2405, e-mail: wuestner@bmb.sdu.dk

Supplemental Information

S1. Analysis of mitochondria length and branching in fibroblasts. Control, NPC1 and NPC2 diseased fibroblasts were incubated with 20 µM CTL, from a CTL/BSA complex for 48 h in LPDS medium and subsequently chased for 24 h in LPDS medium. Some of the NPC2 diseased cells were chased in the presence of 200 nM NPC2 protein. Before imaging were carried out on an UV sensitive wide field microscope, the mitochondria were labeled with MitoTracker Green. Prior to analyzing the length of mitochondria with MiNA, the images were deconvolved as described in *Material and Methods*.

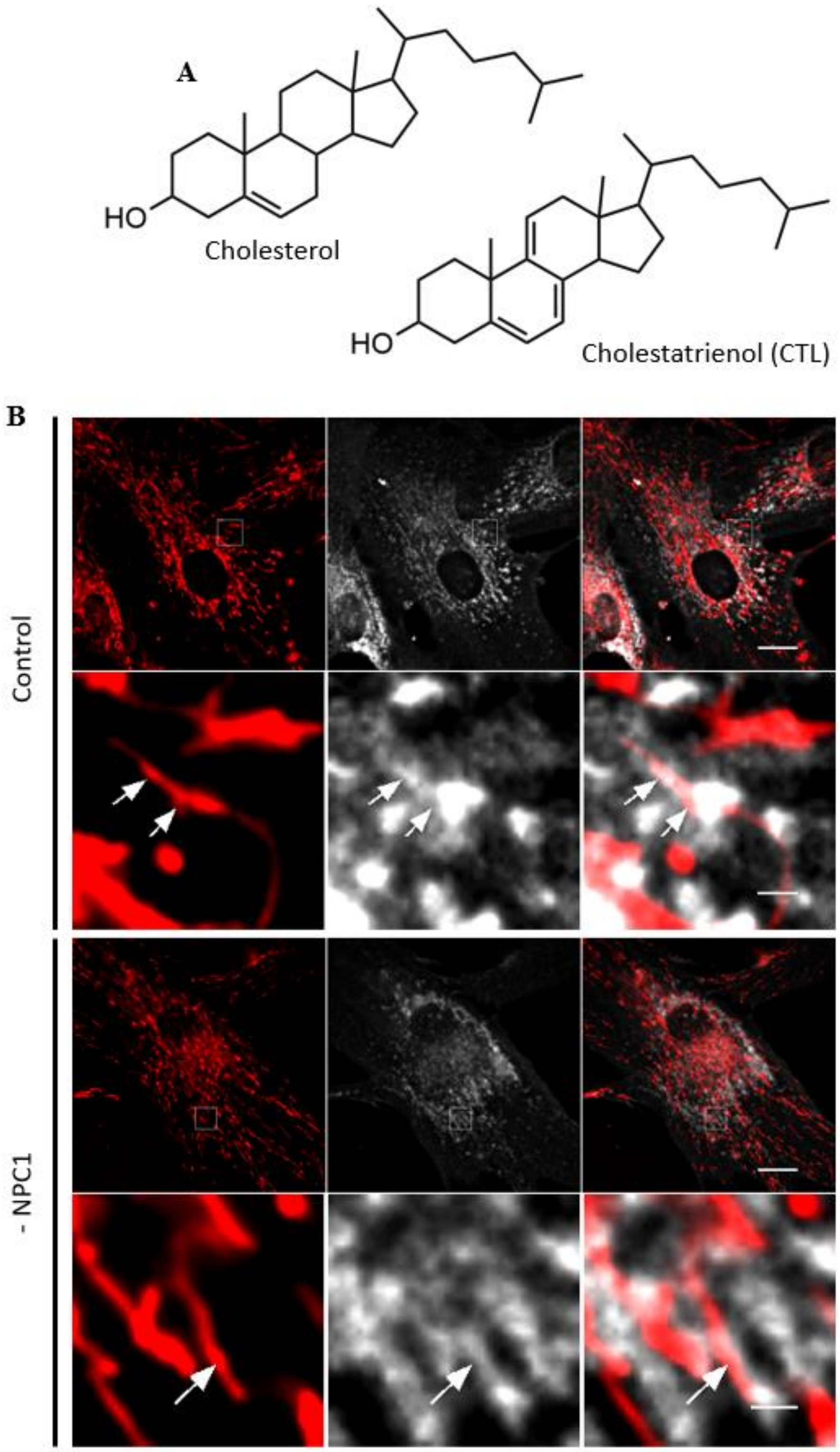

Figure S1. Transport of CTL to mitochondria in control and NPC1-deficient fibroblasts. A, Structure of cholesterol and its fluorescent analogue CTL. Human fibroblasts from a healthy subject ('Control') or from a NPC1-disease patient ('-NPC1') were loaded with 20 µM CTL, from a CTL/BSA complex for 48 h in LPDS medium and subsequently chased for 24 h in LPDS medium, and co-stained with MitoTracker green before imaging on an UV sensitive wide field microscope. Arrows point to CTL in mitochondria. Bar, 20 µm.

A

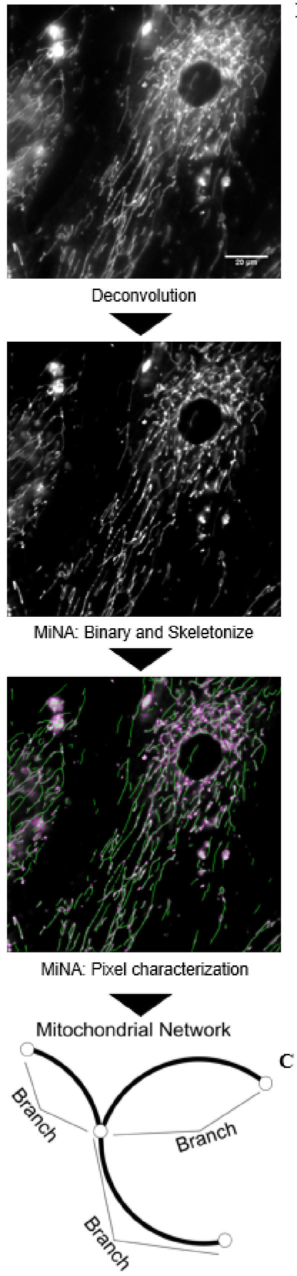

B

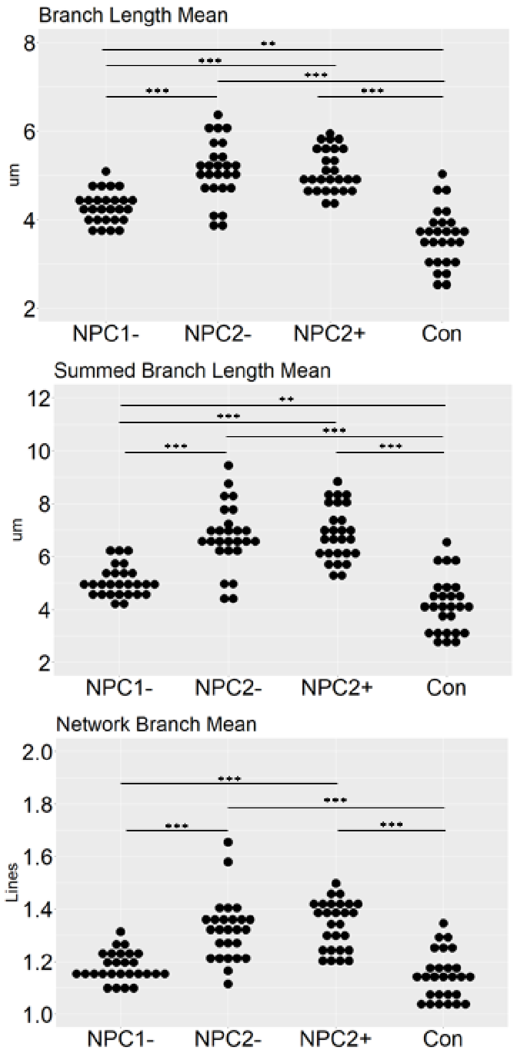

C

Comparaison	BLM P-value	SBLM P-value	NBM P-value
NPC1- vs. con	0.0000817	0.0077190	0.5623113
NPC2- vs. con	0.0000000	0.0000000	0.0000000
NPC2+ vs. con	0.0000000	0.0000000	0.0000000
NPC2- vs. NPC1-	0.0000021	0.0000000	0.0000009
NPC2+ vs. NPC1-	0.0000017	0.0000000	0.0000000
NPC2+ vs. NPC2-	0.9999997	0.9792391	0.8222802

D

Condition	Mean BLM (µm)	Mean SBLM (µm)	Mean NBM (Lines)
NPC1-	4.29 ± 0.35	5.06 ± 0.60	1.18 ± 0.06
NPC2-	5.11 ± 0.67	6.77 ± 1.22	1.32 ± 0.12
NPC2+	5.10 ± 0.48	6.88 ± 1.02	1.34 ± 0.09
Con	3.60 ± 0.64	4.18 ± 1.03	1.15 ± 0.09

Figure S2. Analysis of mitochondrial morphology.
A) Cells were loaded with 200 µL CTL/BSA in 1.8 mL LPDS medium for 48 h and subsequently allowed 24 h chase time in LPDS medium with or without 200 nM NPC protein. Prior to wide field microscopy, the cells were loaded with MitoTracker green.
A) Summarize the workflow in MiNA: the images are first made binary (magenta) and skeletonized (green), before all the pixels within a skeleton are categories. B) Plots of the MiNA results for all 4 cell conditions. *Branch Length Mean* (BLM) is the average of the lengths of all lines used to represent the mitochondrial structures. *Summed Branch Length mean* (SBLM) is the sum of all branch lengths divided by the number of independent networks/ skeletons. Network Branches Mean, is the average number of lines used to represent each structure. N=3 microscope dishes, n=26-27 images for each condition. Significance of differences between groups was assessed with ANOVA followed by Tukey Hones Significant different tests. *P<0.05, **P<0.001, ***P<0.0001. C) Summarizes P-values for B. D) Summarizing table of mean ± standard deviation of the results shown in B.

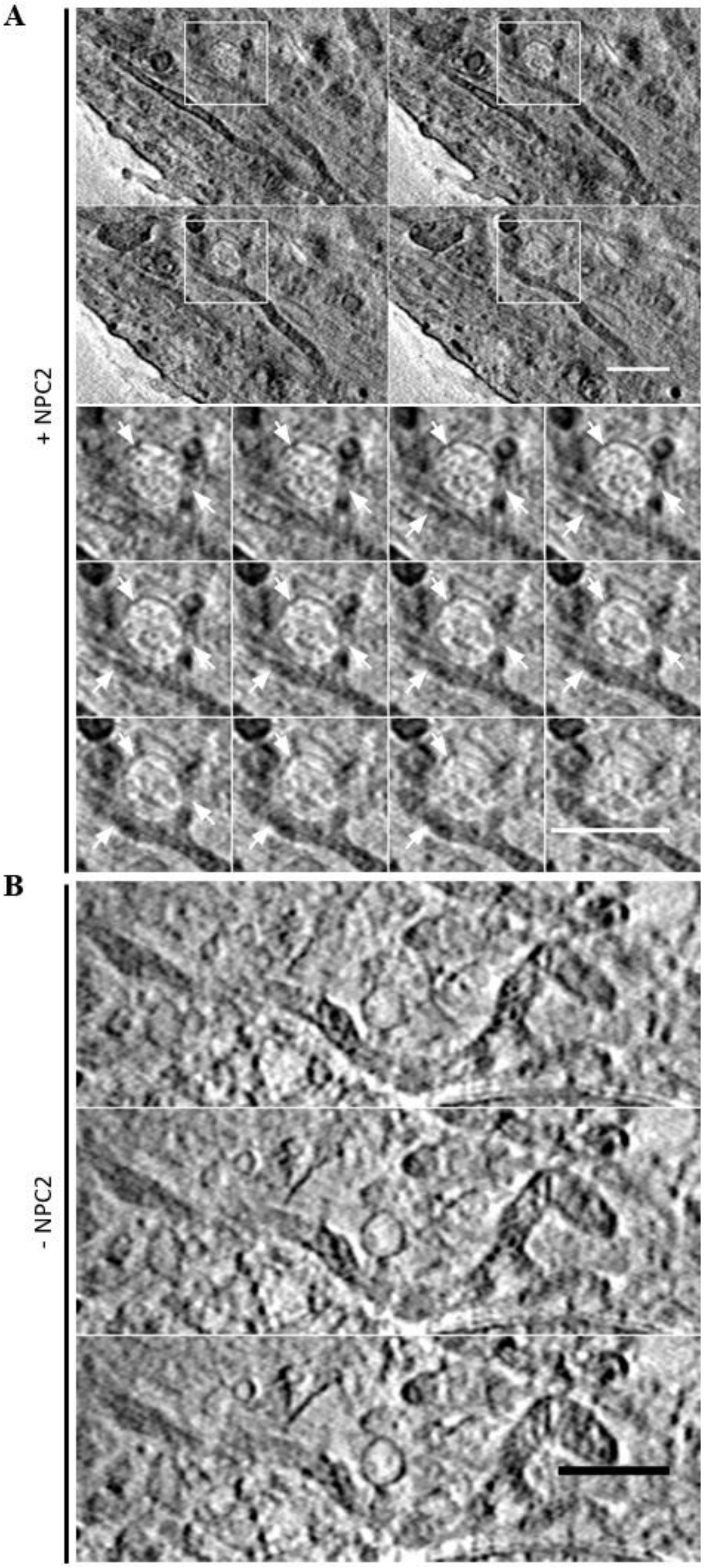

Figure S3. X-ray microscopy of mitochondria ultrastructure in treated and non-treated NPC2-/- cells. NPC2-/- fibroblasts were either treated with 100 nM Alexa546-NPC2 for 72h in LPDS (A) or left untreated in LPDS (B); washed with PBS and fixed with PFA. Cells were kept in PBS until plunge freezing in liquid ethane followed by tomographic imaging at the X-ray microscope, spatial registration and 3D reconstruction of tomograms (see Materials and Methods for details). A, box and zoom highlight a branched mitochondrion embracing a vesicle with intraluminal membrane, suggesting that it is a late endosome or lysosome. B, ultrastructure of NPC2-/- fibroblasts in untreated cells did not differ from that of treated cells. Bar, 1 µm.

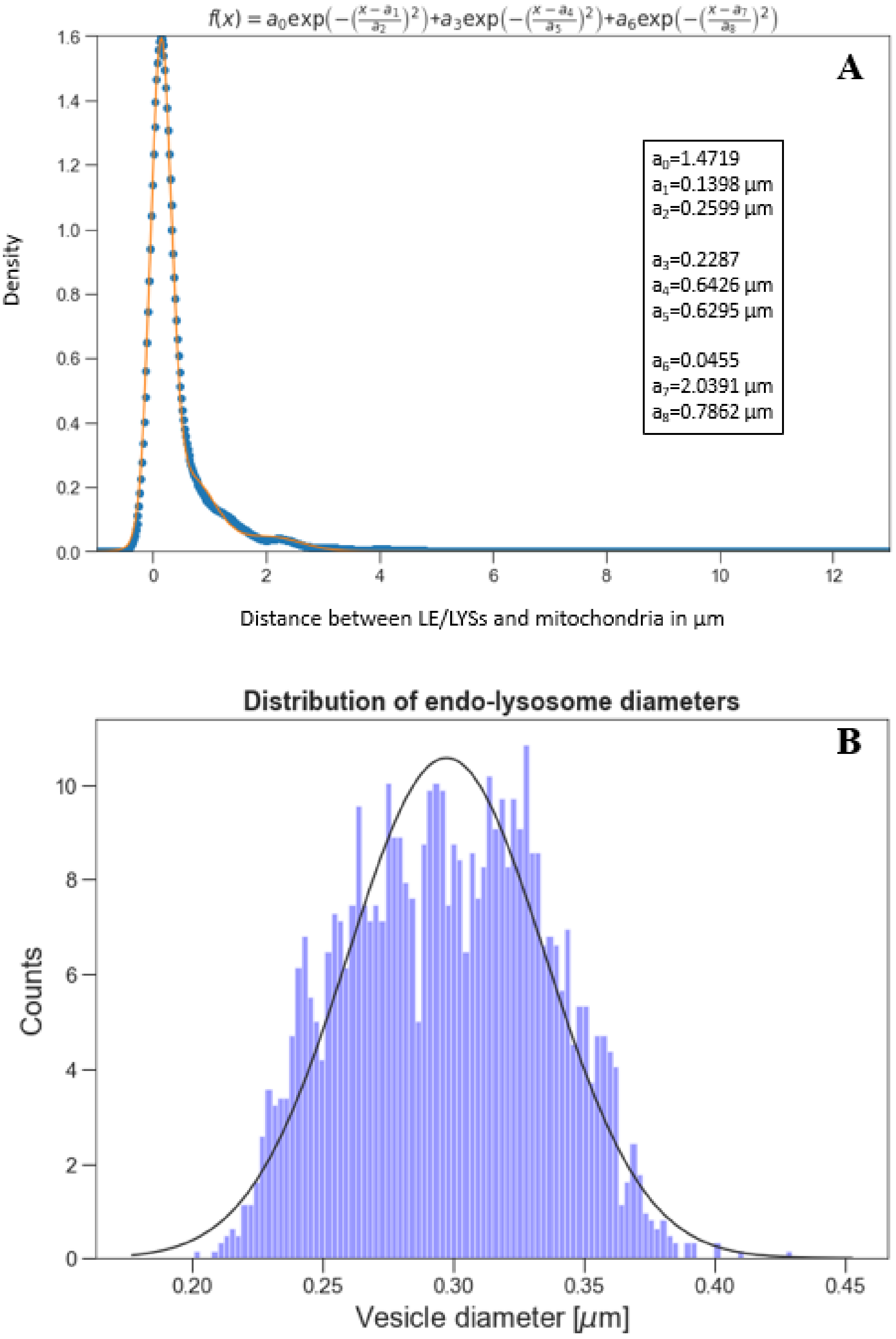

Figure S4. Further analysis of LE/LYSs and their distance to mitochondria.
A, the pooled histogram of measured inter-organelle distance distributions (compare Fig. 3F) was fitted to the sum of three Gaussians with determined parameter values shown as inset. Blue symbols are data, yellow line is the fit. B, histogram of diameters of all LE/LYSs containing Alexa546-NPC2 estimated from five cells using a 2D Gaussian fit to each organelle from 2D images together with a kernel density estimation of the whole population (blue line).

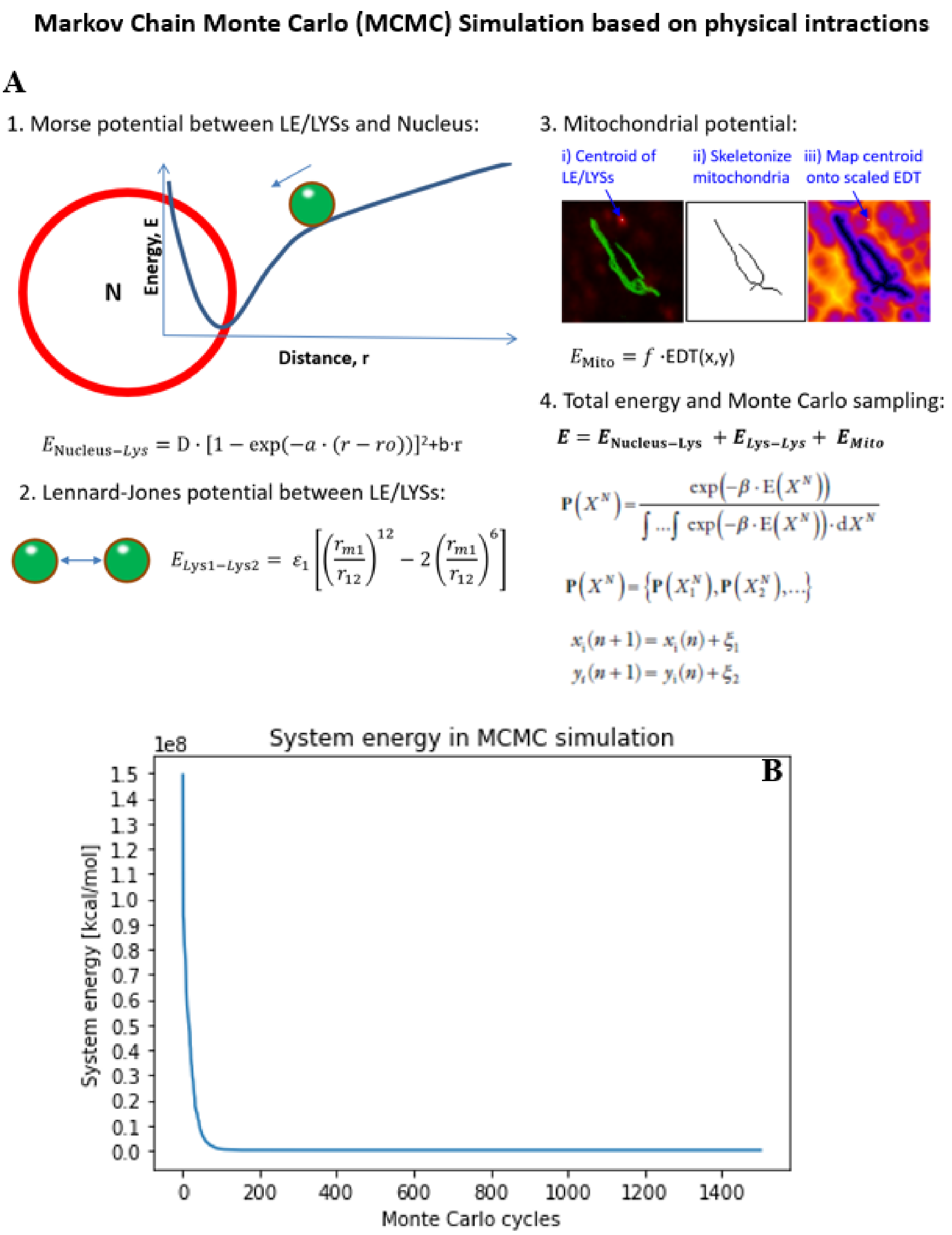

Figure S5. Energy terms of organelle interaction in the Monte Carlo simulation. A, the potential energy describing interactions between endo-lysosomes in fibroblasts consist of three parts; 1. a Morse potential between endo-lysosomes and the nucleus to mimic the attraction of the organelles to the perinuclear region, in cells defined by the location of the microtubule organizing center, 2. an inter-organelle interaction described by a 6,12-Lennard Jones potential and 3. the interaction to mitochondria. This last term is defined based on the Euclidian distance transform of segmented mitochondria. The EDT is defined within the cell geometry and weighted by a factor, f, to get the potential energy landscape. The total energy is given in 4. as sum of these three terms. Particles are randomly moved a distance in x,y and the energy of this new conformation is calculated. The move is accepted with a probability given by the Metropolis criterion, which efficiently samples the whole space of possible configurations along a Markov chain. B, the total energy drops rapidly during the simulation and reaches a plateau value after a few hundred Monte Carlo cycles. This ensures thermodynamic equilibrium during the sampling phase.

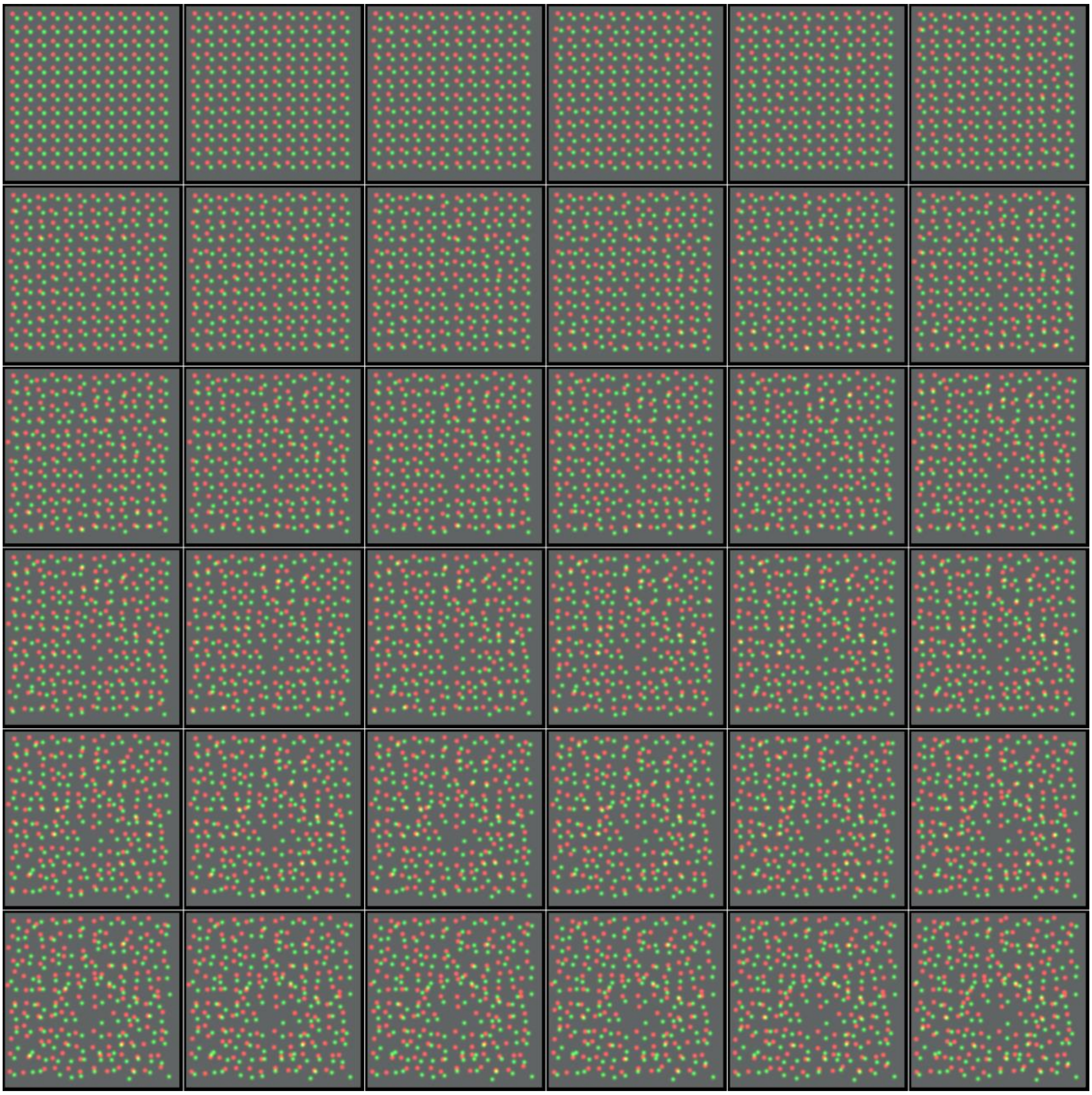

Fig. S6. Snapshots from the two-population MCMC simulation with indicated boundary. The initial 36 configurations of a Monte Carlo simulation are shown to demonstrate that spatial order is rapidly lost, in concert with generation of a particle pattern, which 'avoids' the nucleus due to the Morse potential. Only the last one percent of a simulation comprised of typical 1500 images were used for spatial analysis. They quadratic boundary for particle displacements is shown in grey, but the underlying M-potential given by the mitochondria-derived EDT map is not shown. See Materials and Methods, Figure 3 and Figure S5 for further details.

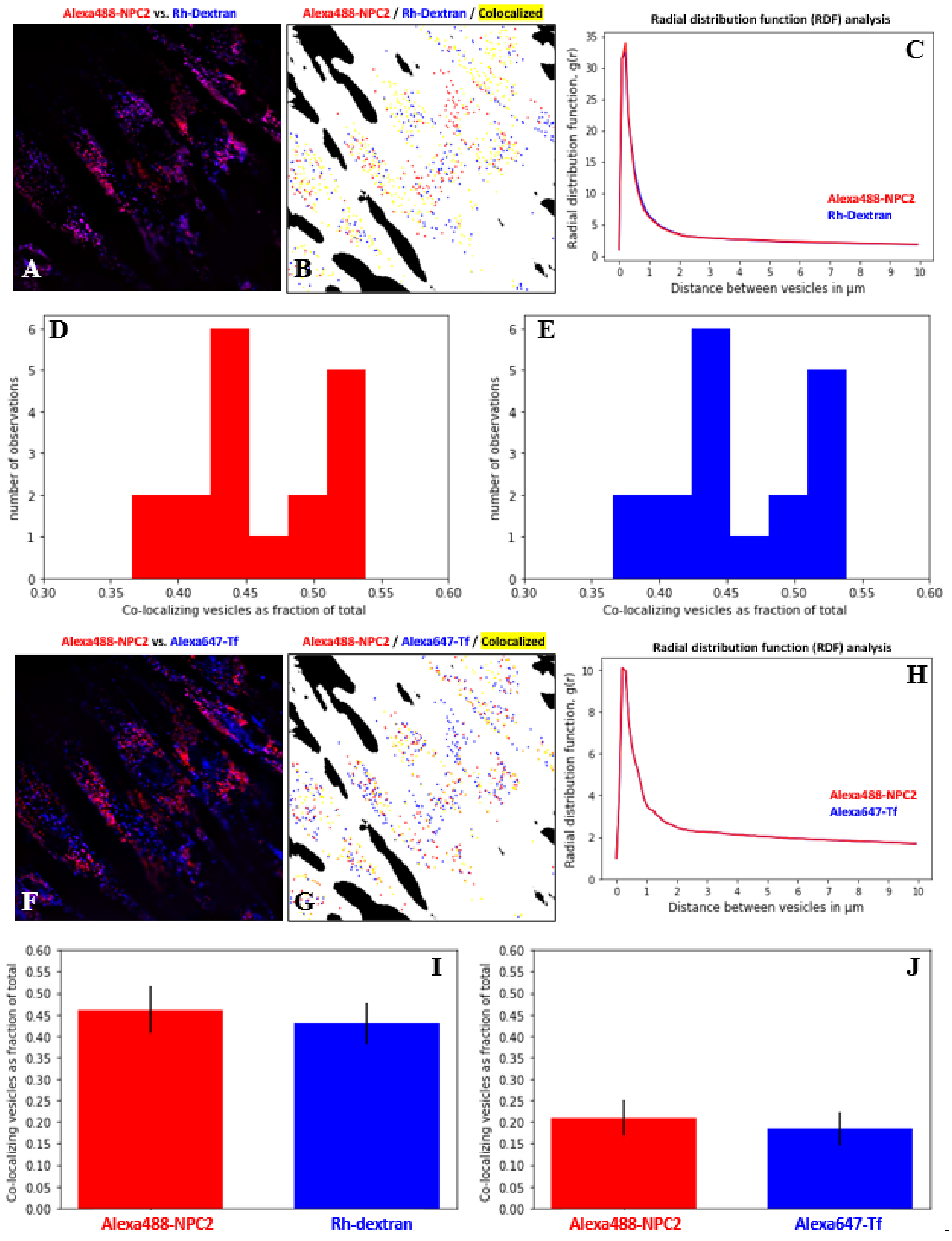

Figure S7. Co-localization analysis of Alexa488-NPC2 with Rh-dextran and Alexa647-Tf. Co-localization analysis was carried for NPC2-deficient fibroblasts labeled with DHE/BSA to detect the cell border, with Alexa488-NPC2, the endo-lysosomal marker Rh-dextran and the early endosome marker Alexa647-Tf. Centroid coordinates and sizes of vesicles were determined by 2D Gaussian fitting. A, color overlay, B, centroid positions of both populations with co-localizing LE/LYSs in yellow. C, radial distribution function (RDF) for the positions identified in B with the reference channel indicated by color. D, E, object-based co-localization of Alexa488-NPC2 vs. Rh-dextran (D) and vice versa (E). F-H, color overlay (F), centroid positions (H) and RDF (H) for fluorescent NPC2 vs. transferrin. Note the lower peak in H compared to C. I, J, fraction of co-localizing vesicles given as mean ± SD of 18 images with 450-650 detected vesicles per channel and image for Alexa488-NPC2 vs. Rh-dextran (I) and vs. Alexa647-Tf (J).

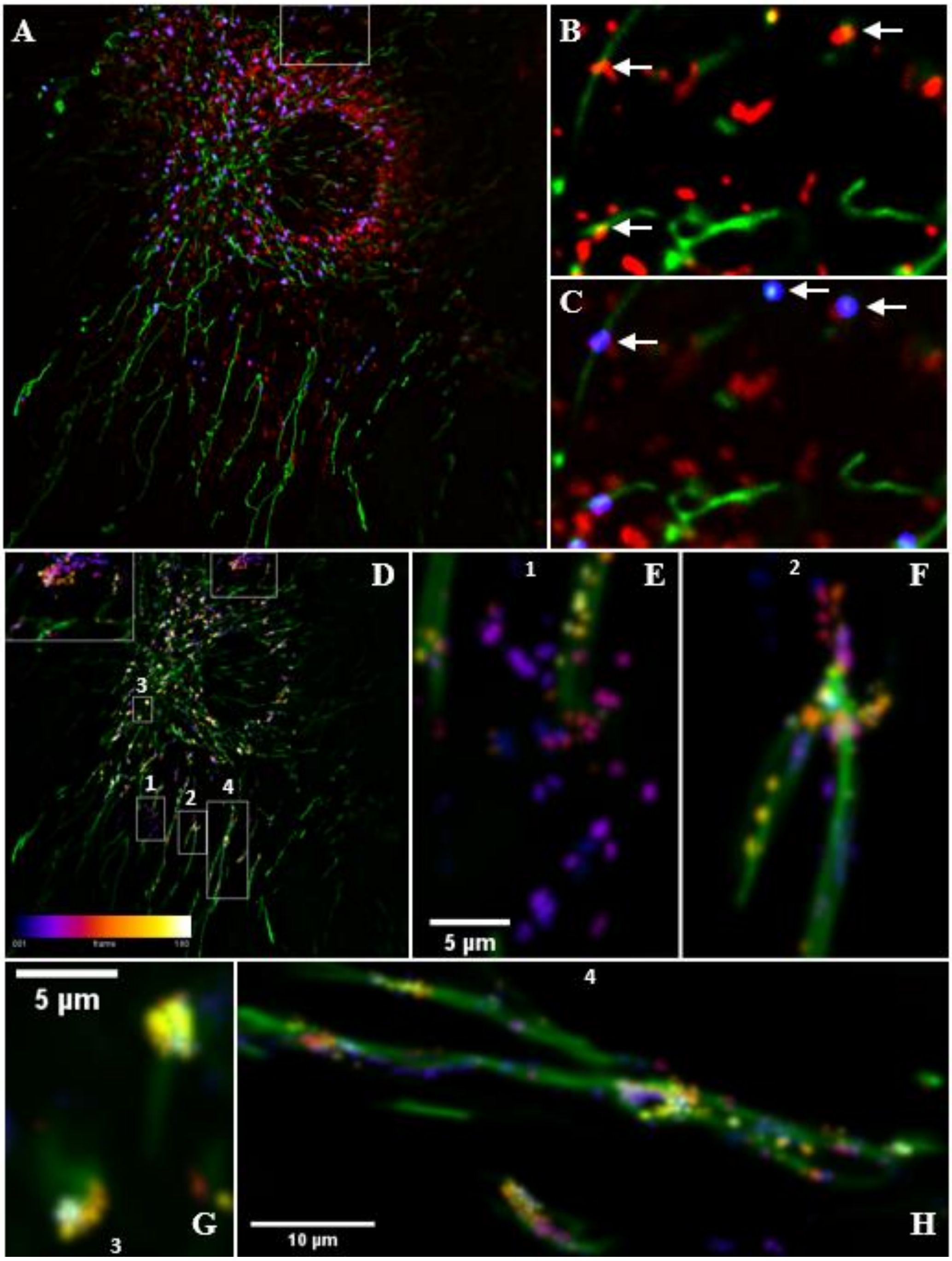

Figure S8. Segmentation of membrane contact sites between endo-lysosomes and mitochondria. Workflow of the analysis is shown in Fig. 8. A, first frame of time-lapse sequence as in Fig. 7A with detected MCSs in blue. Box shown in A is zoomed up prior to segmentation (B) and after segmentation, with identified MCSs in blue (C, arrows point to examples of identified MCSs). D-H, time stamping of identified MCSs overlayed to the mitochondria image. D, overview with zoomed box as inset showing a region with mobile MCSs and color bar with early time points in blue and later time points in yellow to orange. The frame rate is 1 fps. Boxes 1-4 are shown as zoomed panels in E-H.

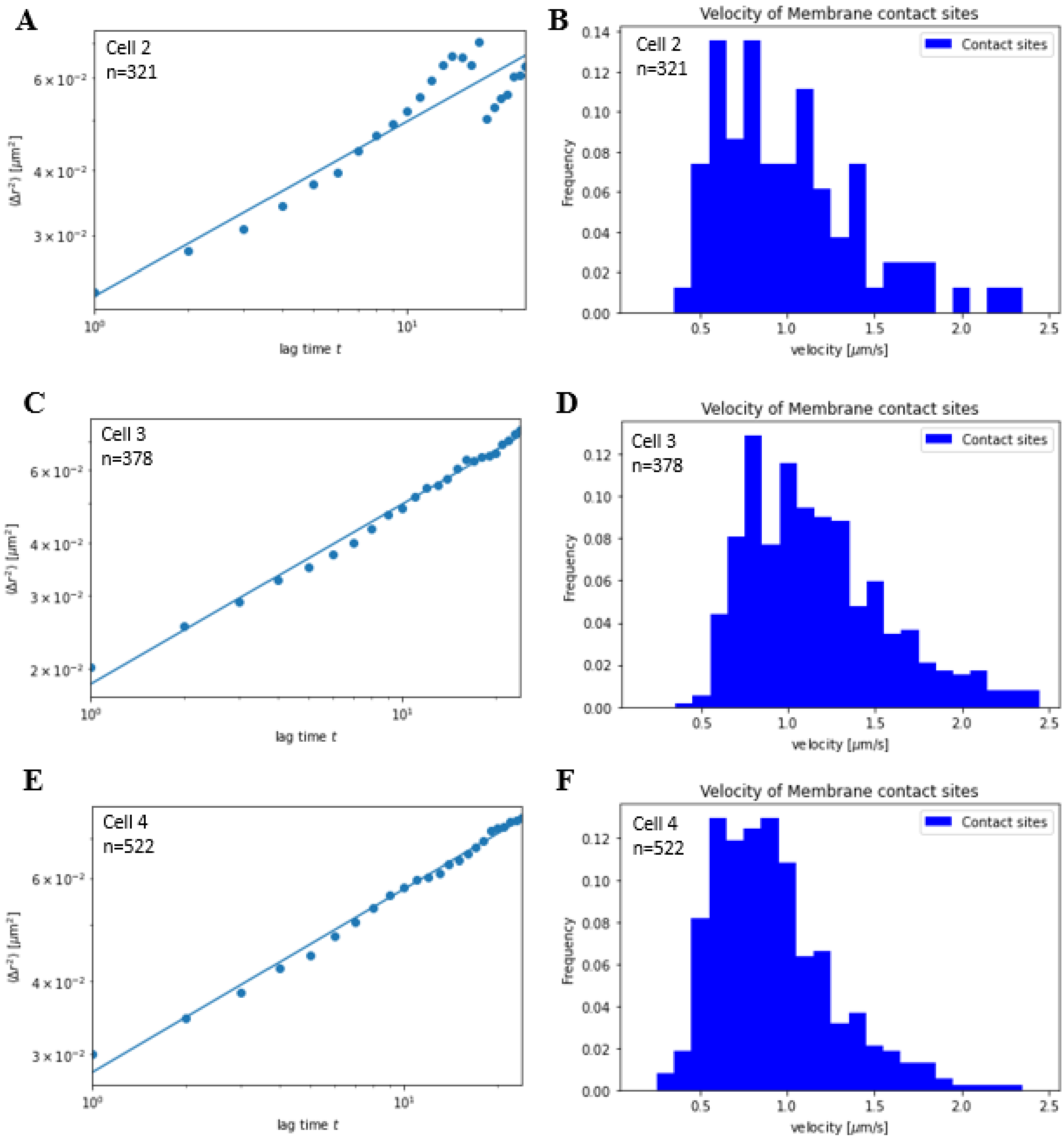

Figure S9. Dynamics of membrane contact sites for additional cells.
The ensemble-averaged mean square displacement MSD was calculated for all trajectories of cells 2 (A), cell 3 (C) and cell 4 (E), with the number of trajectories (n) given in each case. Data was plotted in log-log space and fitted to an anomalous diffusion model, Eq. 1 in Materials and Methods. Histograms of instantaneous velocities of all MCSs tracked for at least 10 sec were calculated and plotted for cell 2 (B), cell 3 (D) and cell 4(F). Compare Figure 10 and main text.

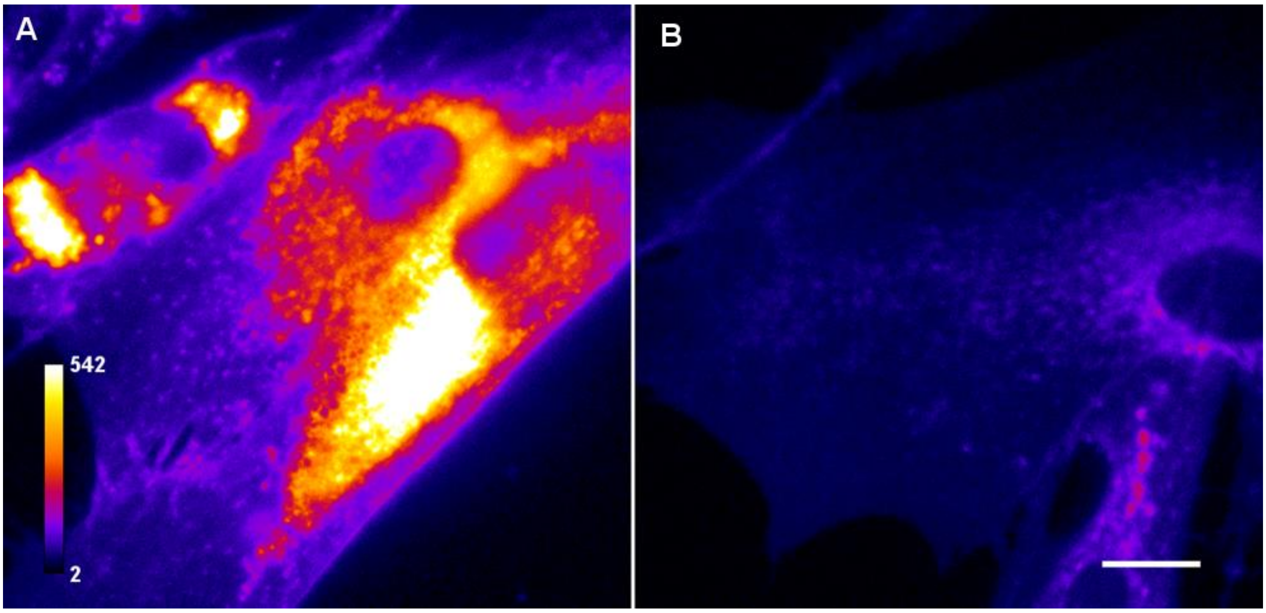

Figure S10. CTL intensity in NPC1-/- and control fibroblasts. Cells were loaded with 200 µL CTL/BSA in 1.8 mL LPDS medium for 48 h and subsequently allowed 24 h chase time in LPDS medium and imaged on a UV-sensitive wide field microscope. NPC1-deficient cells and control cells are shown in A and B, respectively. Bar, 20 µm.

Video legends

For all videos, NPC2-deficient fibroblasts were incubated with 100 nM Alexa546-NPC2 for 72 h and labeled for 30 min with MitoTracker Green before imaging at a spinning disk confocal microscope. Images were acquired every 1 sec for 3 min, while cells were kept at 37° C, 5% CO_2. All videos were compressed as jpeg files in ImageJ before converting to AVI format and are played with 10 frames per second.

Supplemental video 1. Two endo-lysosomes containing Alexa546-NPC2 (red) are in prolonged contact with mitochondria (green) in the lower left and upper right part of the video, respectively. The upper vesicle appears to split into two at a branch point of the mitochondrion.

Supplemental video 2. Fusion of one mitochondrion (green) is observed.

Supplemental video 3. Fission of one mitochondrion (green) into two is observed in the central part of the video.

Supplemental video 4. Endo-lysosomes containing Alexa546-NPC2 (red) glide along mitochondria (green) and keep contact over an extended time period.

Supplemental video 5. Endo-lysosomes containing Alexa546-NPC2 (red) glide perpendicular and along mitochondria (green) and become eventually split into two.

Supplemental video 6. An endo-lysosome containing Alexa546-NPC2 moves at the tip of a branching mitochondrion (green) before it leaves and contacts the second branch of that mitochondrion.

Supplemental video 7. Endo-lysosomes containing Alexa546-NPC2 (red) interact transiently with mitochondrial vesicles (green) as well as with the tip of a mitochondrial tubule, but they don't fuse with mitochondria.

Supplemental video 8. Transient interaction of endo-lysosomes containing Alexa546-NPC2 (red) with mitochondrial vesicles (green) is observed which is accompanied by correlated movement of both organelles and eventual dissociation ('kiss-and-run interaction').

Supplemental video 9. An endo-lysosome containing Alexa546-NPC2 (red) stays in contact with a mitochondrion green for the entire recording of 3 min with very little displacement from its initial position (left part, white box is shown as zoom in the right part). During that time, the endo-lysosome is contacted by other LE/LYSs which seem to fuse with it.
